# Supplementary material for: Diversity in Notch ligand-receptor signaling interactions
Source: bioRxiv. 2023 Aug 25:2023.08.24.554677. Preprint. [Version 1] doi: 10.1101/2023.08.24.554677 (PMC10473737; doi:10.1101/2023.08.24.554677)
Supplement: Supplementary file 1 [file NIHPP2023.08.24.554677v1-supplement-1.pdf]

bioRxiv preprint doi: https://doi.org/10.1101/2023.08.24.554677; this version posted August 25, 2023. The copyright holder for this preprint (which was not certified by peer review) is the author/funder, who has granted bioRxiv a license to display the preprint in perpetuity. It is made available under aCC-BY 4.0 International license.

## Supplementary Figures

bioRxiv preprint doi: https://doi.org/10.1101/2023.08.24.554677; this version posted August 25, 2023. The copyright holder for this preprint (which was not certified by peer review) is the author/funder, who has granted bioRxiv a license to display the preprint in perpetuity. It is made available under aCC-BY 4.0 International license.

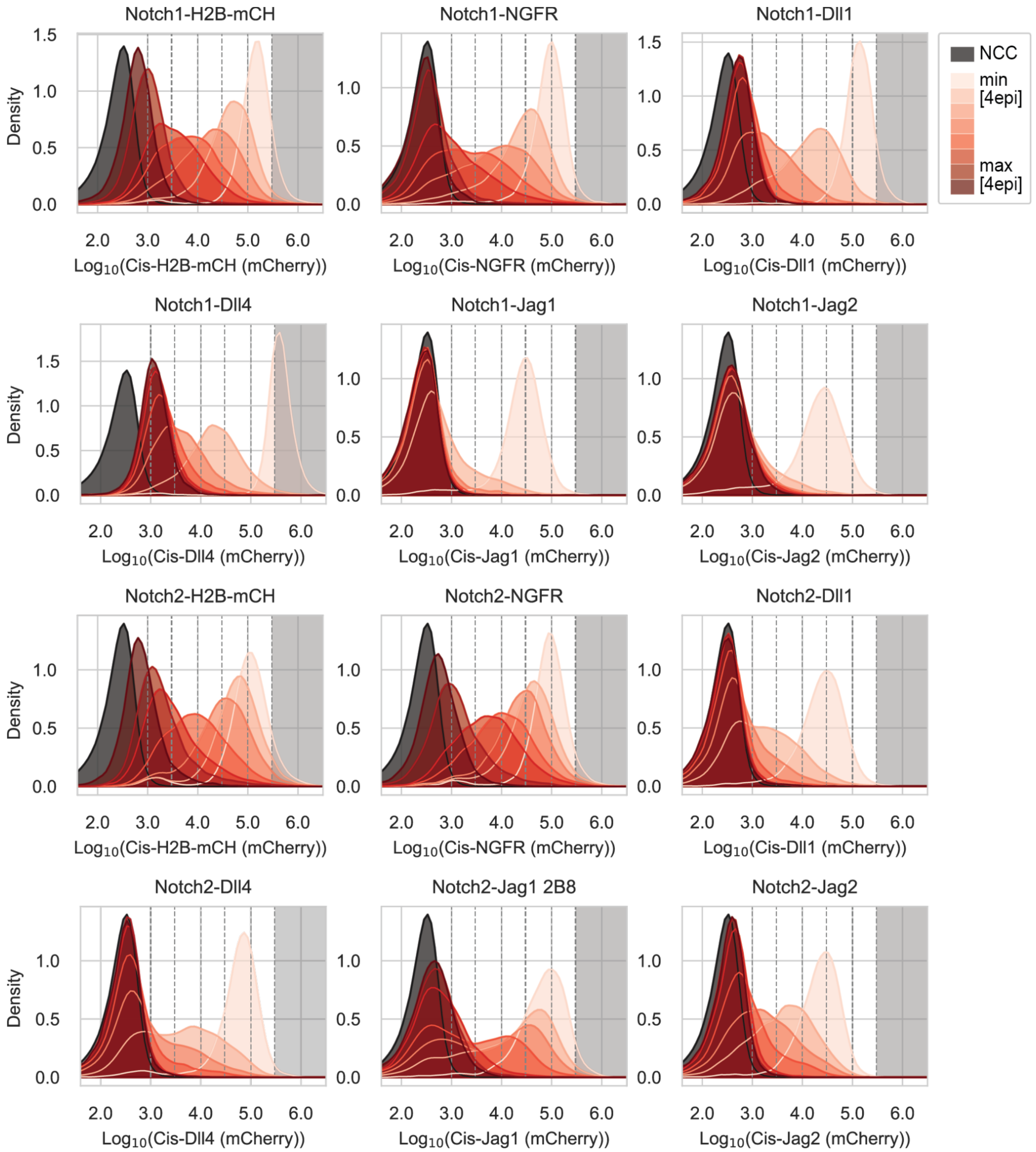


**Figure 1—figure supplement 1: The Tet-OFF system enables unimodal titration of ligand levels in receiver cells**

Distributions of cotranslational cis-ligand expression (mCherry, A.U.) in the receiver cell lines used to generate data in Figure 4. Each histogram contains data from three biological replicates corresponding to a specific 4-epi-Tc concentration ranging from 0-500 ng/mL (Supplementary Table 3). Black histograms are CHO-K1 wild-type cells (NCC = no color control). Vertical dashed

bioRxiv preprint doi: https://doi.org/10.1101/2023.08.24.554677; this version posted August 25, 2023. The copyright holder for this preprint (which was not certified by peer review) is the author/funder, who has granted bioRxiv a license to display the preprint in perpetuity. It is made available under aCC-BY 4.0 International license.

lines indicate mCherry bins used to generate dose response curves elsewhere (Methods). Datapoints in the grey-shaded region were discarded due to overexpression artifacts at high levels.

bioRxiv preprint doi: https://doi.org/10.1101/2023.08.24.554677; this version posted August 25, 2023. The copyright holder for this preprint (which was not certified by peer review) is the author/funder, who has granted bioRxiv a license to display the preprint in perpetuity. It is made available under aCC-BY 4.0 International license.

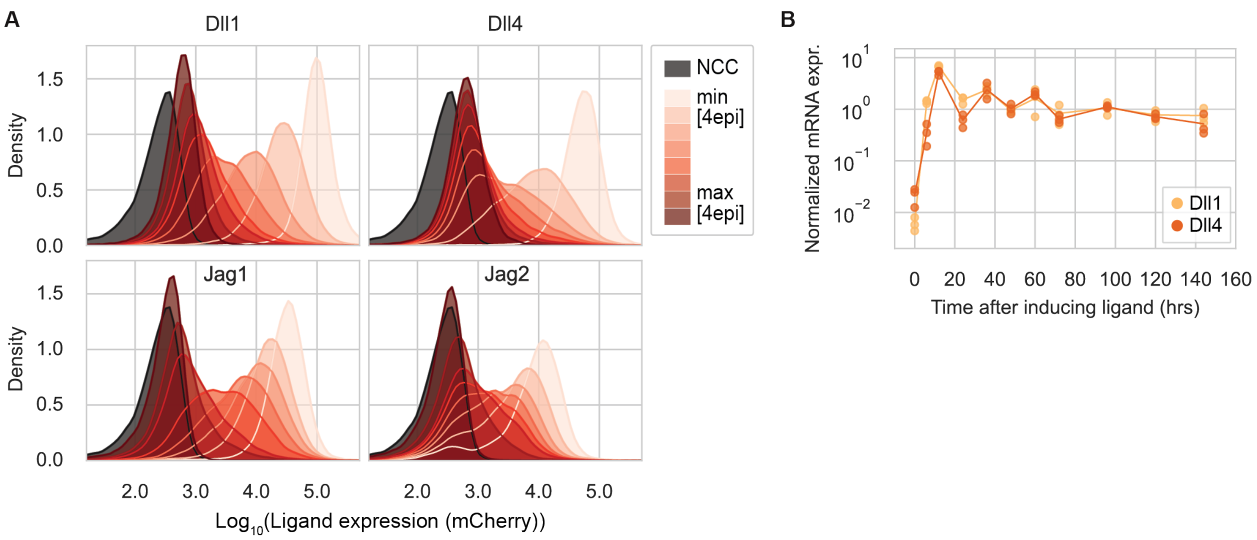


**Figure 1—figure supplement 2: The Tet-OFF system enables stable ligand expression in sender cells**

(**A**) Distributions of cotranslational ligand expression (mCherry, A.U.) in Tet-OFF sender cell ines expressing each of the four activating Notch ligands, tuned to different expression levels via titration of 4-epi-Tc concentration. Each histogram contains data from three biological replicates. Black histograms are wild-type CHO-K1 (NCC = no color control).

(**B**) mRNA expression from the Tet-OFF promoter reaches stable levels after 24-48 hours. CHO-K1 senders with integrated Dll1 (yellow) or Dll4 (orange) ligands driven by the Tet-OFF promoter were induced to express ligand by reducing [4-epi-Tc] in the culture medium at time zero. Y-axis values are $2^{-\Delta Cq}$ values quantifying mCherry transcript levels at the indicated time of cell harvest (x-axis) relative to the housekeeping gene beta-actin, normalized to the average of all data points after 36 hr for each ligand individually. Three biological replicates were collected for each cell type and time point.

bioRxiv preprint doi: https://doi.org/10.1101/2023.08.24.554677; this version posted August 25, 2023. The copyright holder for this preprint (which was not certified by peer review) is the author/funder, who has granted bioRxiv a license to display the preprint in perpetuity. It is made available under aCC-BY 4.0 International license.

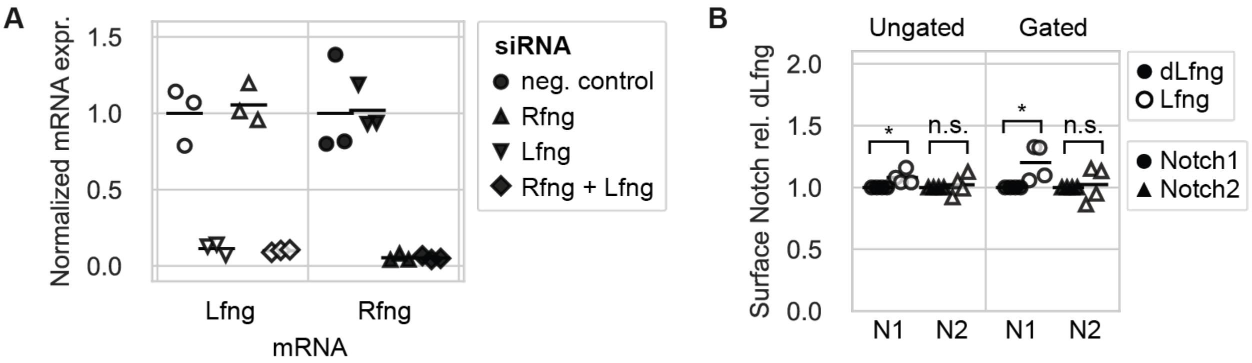


**Figure 1—figure supplement 3: Knockdown of endogenous Lfng and Rfng in CHO-K1 cells**

(**A**) qRT-PCR analysis of wild-type CHO-K1 Lfng and Rfng transcripts from cells treated with either a negative control siRNA, Rfng siRNA, Lfng siRNA, or Rfng and Lfng siRNAs together. The siRNA used is indicated by marker shape as shown in the legend while each column corresponds to an mRNA transcript assayed by qRT-PCR: Lfng (left, open symbols) and Rfng (right, closed symbols). Y-axis values are $2^{-\Delta Cq}$ values (computed using beta-actin as a housekeeping gene) normalized to the expression level in the negative control knockdown for each gene. Black bars are the mean of three biological replicates.

(**B**) Log2 fold-difference in the amount of surface Notch detected by staining with PE-conjugated antibodies (median PE fluorescence (A.U.) from single-cell flow cytometry data) in different Fringe expression conditions relative to Notch levels with dLfng expression (normalized within each bioreplicate). The receptor is labeled on the x-axis (where "N" stands for "Notch") and denoted by marker shape. Marker fill represents the Fringe construct transfected along with an IFP2 cotransfection marker. Asterisk-labeled brackets label data sets unlikely ($p$-val < 0.05) to come from distributions with the same means according to permutation testing with 10,000 bootstrap replicates (Methods). Horizontal bars are mean of four biological replicates.

bioRxiv preprint doi: https://doi.org/10.1101/2023.08.24.554677; this version posted August 25, 2023. The copyright holder for this preprint (which was not certified by peer review) is the author/funder, who has granted bioRxiv a license to display the preprint in perpetuity. It is made available under aCC-BY 4.0 International license.

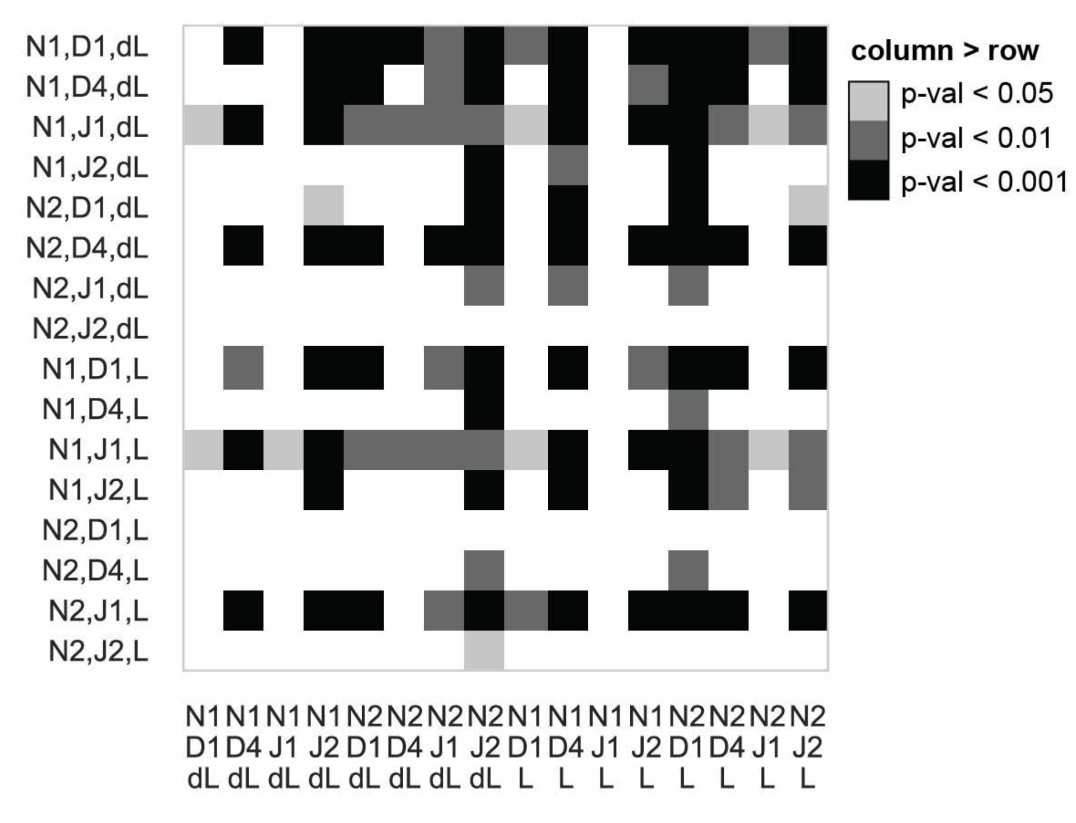


**Figure 2—figure supplement 1: Statistical analysis of differences in trans-activation strength for all receptor-ligand-Fringe combinations**

A matrix of p-values computed by comparing the relative strengths of 64 receptor-ligand-Fringe combinations from data in Figure 2A. For each matrix entry (row, column), the p-value was computed from testing the alternative hypothesis that the Notch combination in the column came from a distribution with a lower mean than the Notch combination in the corresponding row. Thus, dark squares indicate that the Notch combination labeling its column is likely to signal more strongly than the Notch combination labeling its row. P-values were computed via permutation testing with 10,000 bootstrap replicates (Methods).

bioRxiv preprint doi: https://doi.org/10.1101/2023.08.24.554677; this version posted August 25, 2023. The copyright holder for this preprint (which was not certified by peer review) is the author/funder, who has granted bioRxiv a license to display the preprint in perpetuity. It is made available under aCC-BY 4.0 International license.

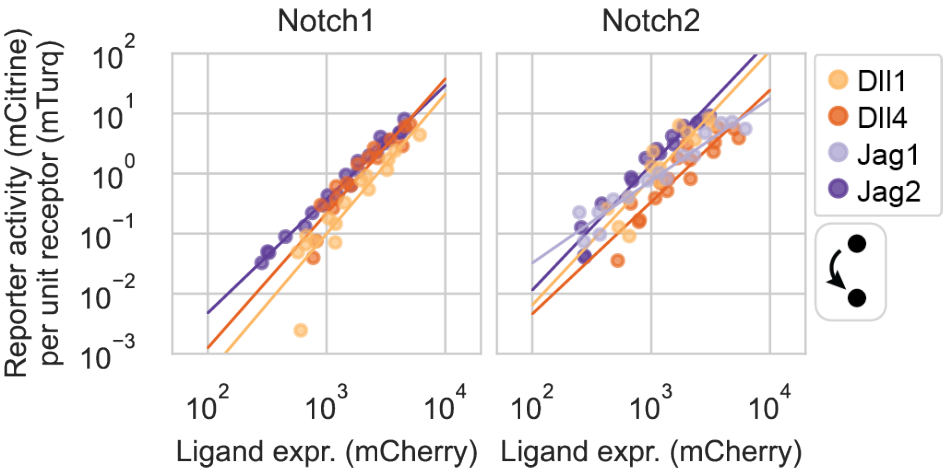


**Figure 2—figure supplement 2: Notch trans-activation in coculture is modestly ultrasensitive**

Linear regressions to log-log dose response curves from Figure 2D (trans-activation assay), in the sub-saturating range (Methods). X-axis values are the mean of the mCherry fluorescence in the sender cells. Y-axis values are the mean signaling activity, mCitrine (reporter activity, A.U.) divided by mTurq2 (cotranslational receptor expression, A.U.), in receiver cells cocultured with the senders.

bioRxiv preprint doi: https://doi.org/10.1101/2023.08.24.554677; this version posted August 25, 2023. The copyright holder for this preprint (which was not certified by peer review) is the author/funder, who has granted bioRxiv a license to display the preprint in perpetuity. It is made available under aCC-BY 4.0 International license.

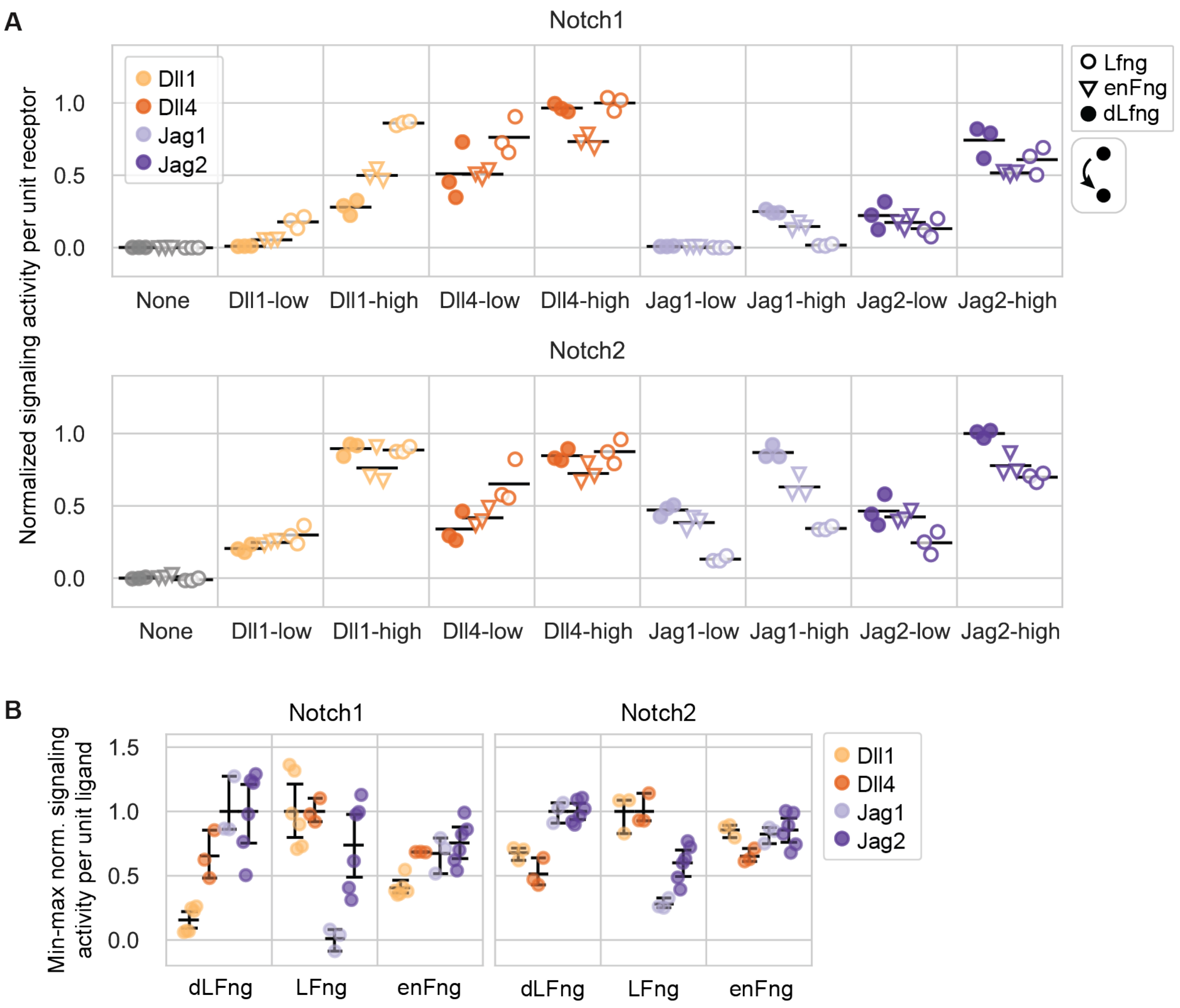


**Figure 2—figure supplement 3: Endogenous Lfng activity dominates over Rfng in CHO-K1 cells**

(**A**) Comparison of ligand-receptor signaling strengths in a trans-activation assay with CHO-K1 Notch1 and Notch2 receivers expressing endogenous CHO-K1 Fringes ("enFng"), dLfng, or Lfng (see Methods). Sender populations used in each coculture are given on the x-axis. For ligand expression distributions, see Supplementary Table 4 and Figure 1C. Y-axis signaling activity values are reporter activity (mCitrine, A.U.) divided by cotranslational receptor expression (mTurq2, A.U.), averaged across receivers, background subtracted, and normalized to the strongest signaling activity measured for each receiver clone in this experiment. Black bars are the mean of three biological replicates.

(**B**) Subsaturating signaling activities from (A) were further normalized to the mean expression of the sender population (Figure 1C) cocultured with each receiver as in Figure 2A (Methods). The ligand-normalized signaling activities were normalized for each receiver such that the bioreplicate

bioRxiv preprint doi: https://doi.org/10.1101/2023.08.24.554677; this version posted August 25, 2023. The copyright holder for this preprint (which was not certified by peer review) is the author/funder, who has granted bioRxiv a license to display the preprint in perpetuity. It is made available under aCC-BY 4.0 International license.

mean of each ligand's strongest Fringe condition is equal to 1. Black bars are mean and 95% confidence intervals from 10,000 bootstrap replicates.

bioRxiv preprint doi: https://doi.org/10.1101/2023.08.24.554677; this version posted August 25, 2023. The copyright holder for this preprint (which was not certified by peer review) is the author/funder, who has granted bioRxiv a license to display the preprint in perpetuity. It is made available under aCC-BY 4.0 International license.

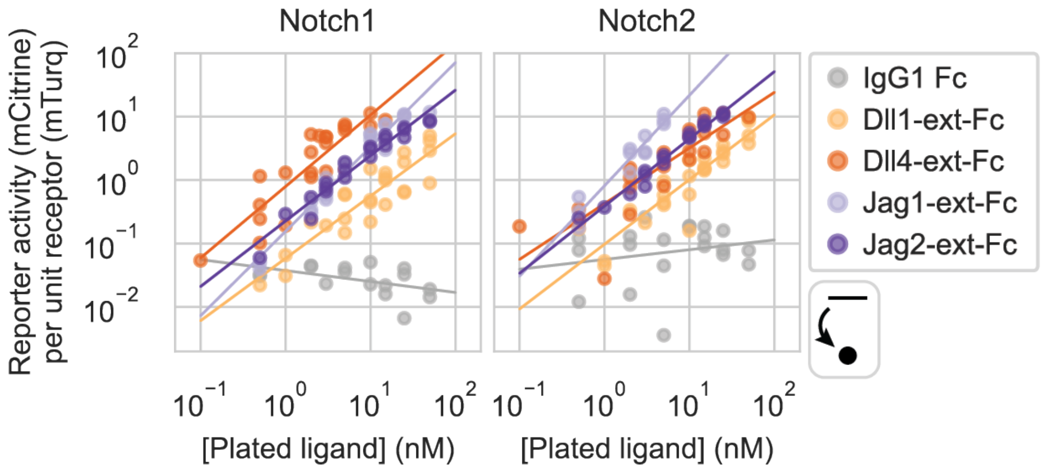


**Figure 3—figure supplement 1: Notch activation by plated ligands is not ultrasensitive**

Linear regressions to log-log dose response curves from Figure 3B (plated ligand assay), in the sub-saturating range. Y-axis signaling activity values are the mean of the distribution of mCitrine (reporter activity, A.U.) divided by mTurq2 (cotranslational receptor expression, A.U.).

bioRxiv preprint doi: https://doi.org/10.1101/2023.08.24.554677; this version posted August 25, 2023. The copyright holder for this preprint (which was not certified by peer review) is the author/funder, who has granted bioRxiv a license to display the preprint in perpetuity. It is made available under aCC-BY 4.0 International license.

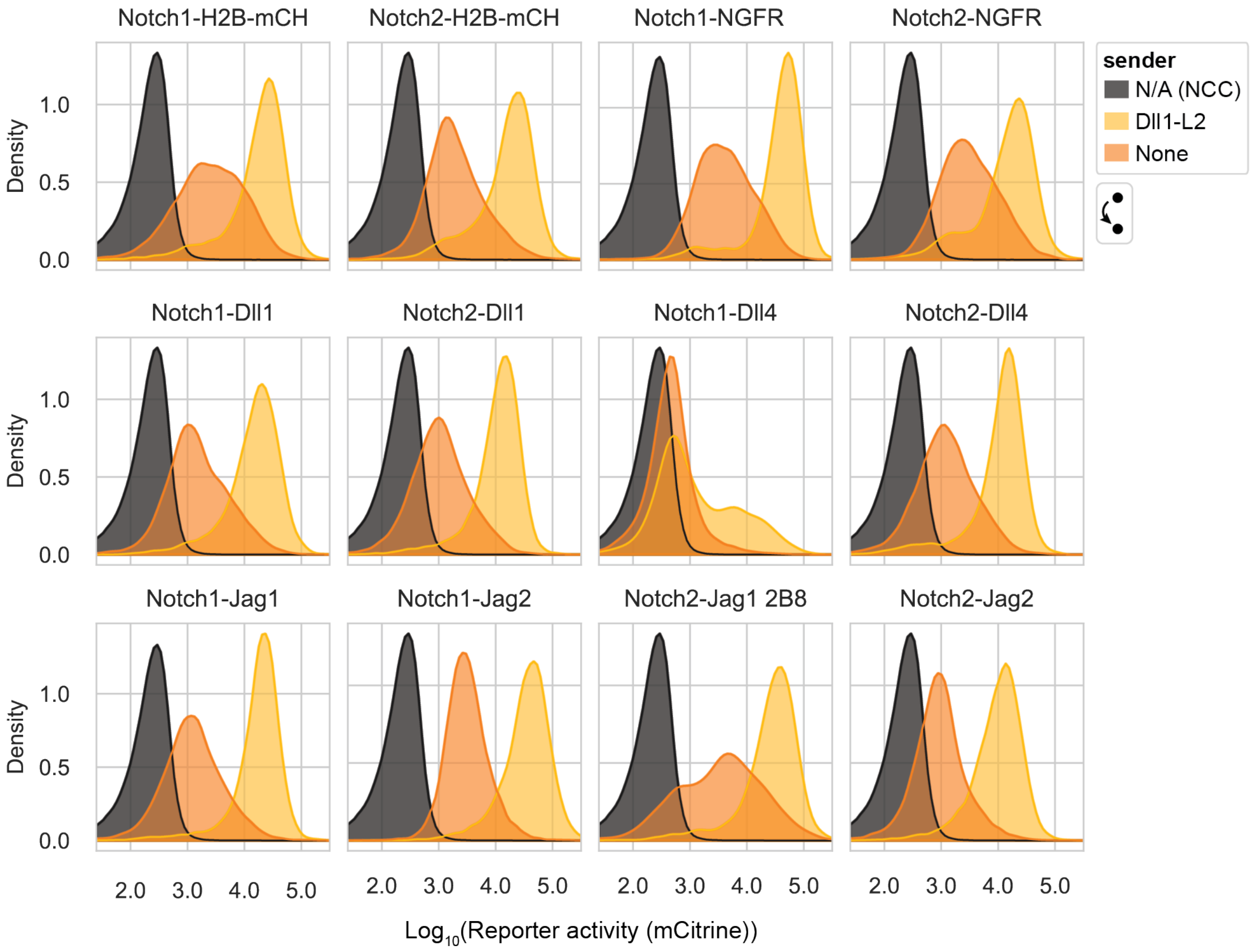


**Figure 4—figure supplement 1: CHO-K1 receiver clones' reporter dynamic ranges with minimum cis-ligand expression**

Histograms of reporter activity (mCitrine, A.U.) in the indicated receiver cells with minimum cis-ligand (maximum [4-epi-Tc]), cocultured with wild-type CHO-K1 cells (sender = "None") or high-Dll1 CHO-K1 senders. Each receiver histogram includes pooled data from three biological replicates.

bioRxiv preprint doi: https://doi.org/10.1101/2023.08.24.554677; this version posted August 25, 2023. The copyright holder for this preprint (which was not certified by peer review) is the author/funder, who has granted bioRxiv a license to display the preprint in perpetuity. It is made available under aCC-BY 4.0 International license.

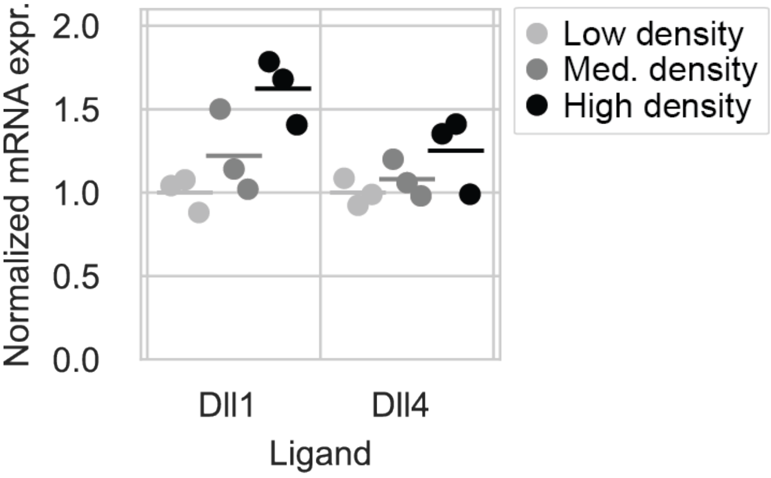


**Figure 4—figure supplement 2: Cell density can affect expression from the Tet-OFF promoter**

Cell density can affect Tet-OFF controlled ligand expression. 25,000 CHO-K1 Dll1 or Dll4 sender cells were cultured at three different densities and induced to maximal ligand expression by removing 4-epi-Tc from the culture medium (Methods). Cells were collected 72 hr later and RNA expression was analyzed by qRT-PCR. Y-axis values were computed as in Figure 1—figure supplement 2B, except here they were normalized to transcript levels in the 'low' density condition. Horizontal bars are the mean of three biological replicates.

bioRxiv preprint doi: https://doi.org/10.1101/2023.08.24.554677; this version posted August 25, 2023. The copyright holder for this preprint (which was not certified by peer review) is the author/funder, who has granted bioRxiv a license to display the preprint in perpetuity. It is made available under aCC-BY 4.0 International license.

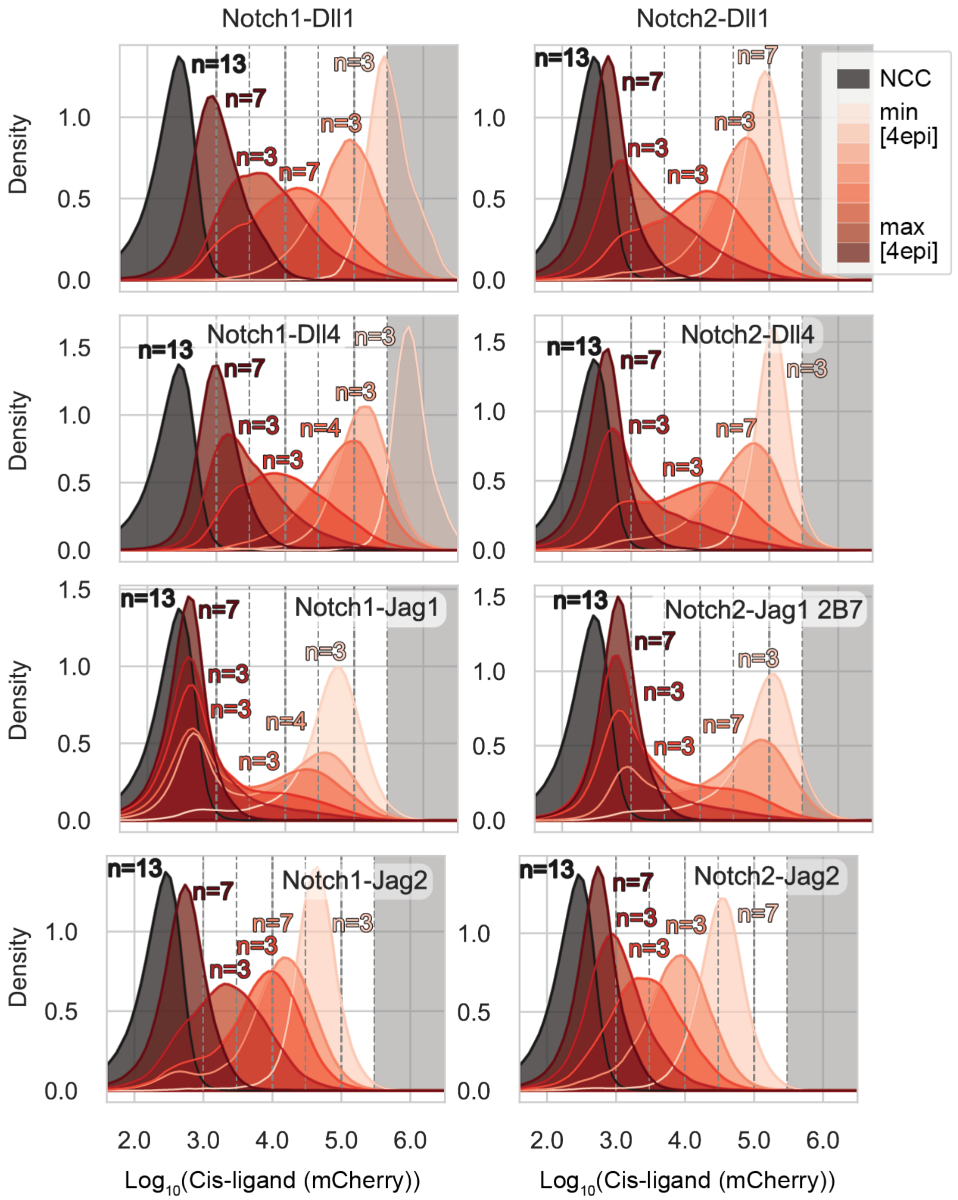


**Figure 5—figure supplement 1: CHO-K1 receiver clones' cis-ligand expression distributions for the cis-activation assay and cis- + trans-activation assay**

Distributions of cotranslational cis-ligand expression (mCherry, A.U.) corresponding to receivers used to generate data in Figure 5A. Each histogram corresponds to a specific 4-epi-Tc concentration ranging from 0-500 ng/mL, and includes data from the indicated number of biological replicates (*n*). Black histograms are CHO-K1 wild-type cells (NCC = no color control). To generate Figure 5A curves, receiver cells were binned into discrete intervals of mCherry expression indicated by the vertical dashed lines. Datapoints in the grey-shaded region were discarded to avoid overexpression artifacts (Methods).

bioRxiv preprint doi: https://doi.org/10.1101/2023.08.24.554677; this version posted August 25, 2023. The copyright holder for this preprint (which was not certified by peer review) is the author/funder, who has granted bioRxiv a license to display the preprint in perpetuity. It is made available under aCC-BY 4.0 International license.

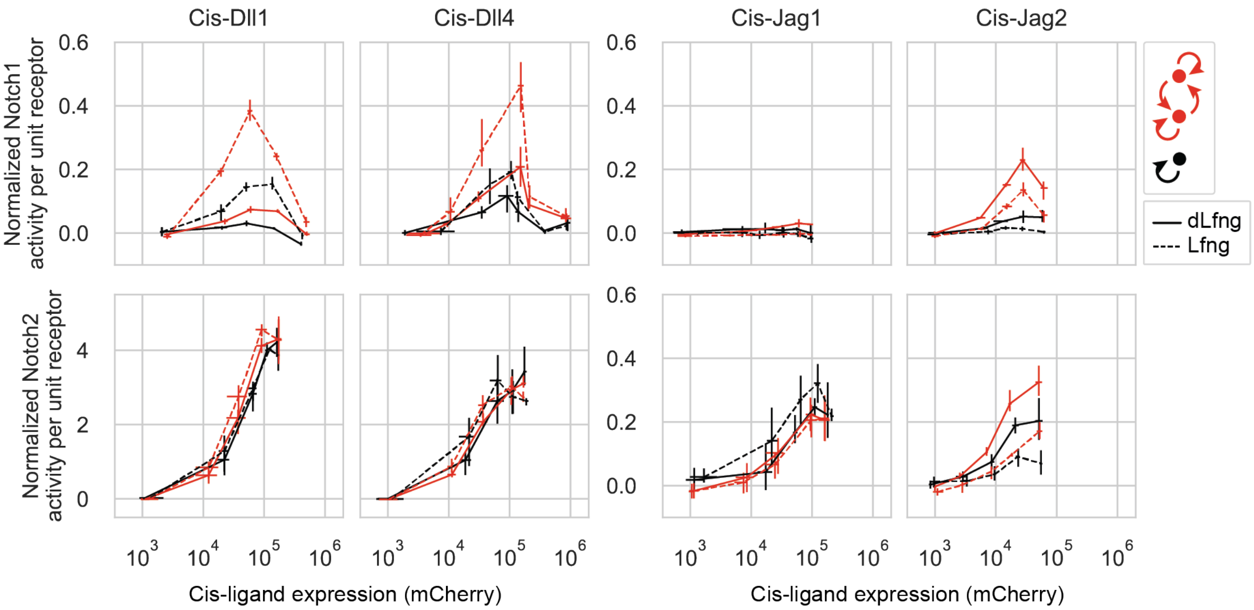


**Figure 5—figure supplement 2: Flow cytometry data analysis pipeline without mCherry binning yields similar results**

Results of cis-activation (black) and cis- + trans-activation (red) assays for CHO-K1 Notch1 (top row) and Notch2 (bottom row) receivers coexpressing a Notch ligand at different levels corresponding to a range of 4-epi-Tc concentrations. These are the same data shown in Figure 5A, but instead of averaging fluorescence distributions across cells binned into different mCherry levels, distributions were averaged across all cells corresponding to the same 4-epi-Tc concentration. Line type denotes expression of dLfng (solid) or Lfng (dashed). Y-axis values are min-max normalized average signaling activities (mCitrine (reporter activity, A.U.) divided by mTurq2 (cotranslational receptor expression, A.U.)). Lines connect means of seven biological replicates in each mCherry bin. Error bars are 95% confidence intervals on the mean value across 10,000 bootstrap replicates.

bioRxiv preprint doi: https://doi.org/10.1101/2023.08.24.554677; this version posted August 25, 2023. The copyright holder for this preprint (which was not certified by peer review) is the author/funder, who has granted bioRxiv a license to display the preprint in perpetuity. It is made available under aCC-BY 4.0 International license.

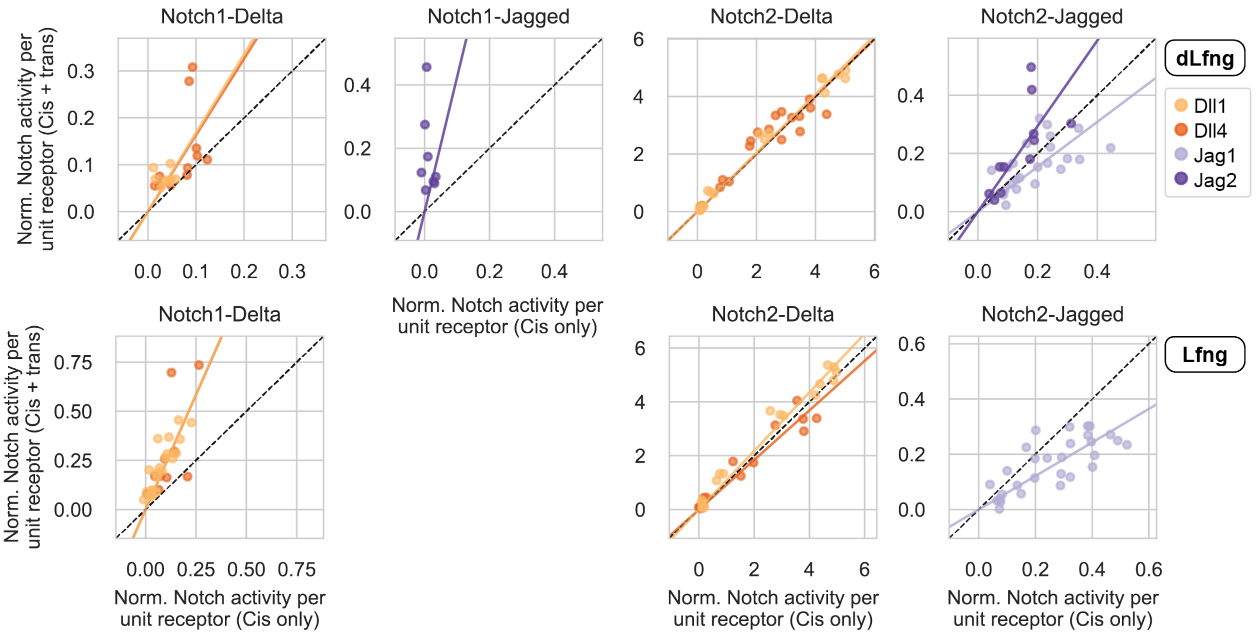


**Figure 5—figure supplement 3: Allowing intercellular contacts between cells coexpressing ligands and receptors alters signaling activity**

Scatterplots comparing normalized signaling activities in the cis- + trans-activation assay (y-axis) with those in the cis-activation assay (x-axis) for receivers expressing dLfng (top row) or Lfng (bottom row). Normalized signaling activities are identical to those in Figure 5A but include only the increasing phase for biphasic curves. Signaling activities below a signaling threshold of 0.05 were also excluded. Ligand-receptor combinations with fewer than five datapoints above the minimum signaling threshold were omitted. Each plot contains curves for one receptor and both cis-ligands of the same class (Delta or Jagged), and color indicates the cis-ligand for each curve. Solid lines are least-squares best-fits, and the black dashed line is y=x. See bootstrapped slope estimates in Figure 5B.

bioRxiv preprint doi: https://doi.org/10.1101/2023.08.24.554677; this version posted August 25, 2023. The copyright holder for this preprint (which was not certified by peer review) is the author/funder, who has granted bioRxiv a license to display the preprint in perpetuity. It is made available under aCC-BY 4.0 International license.

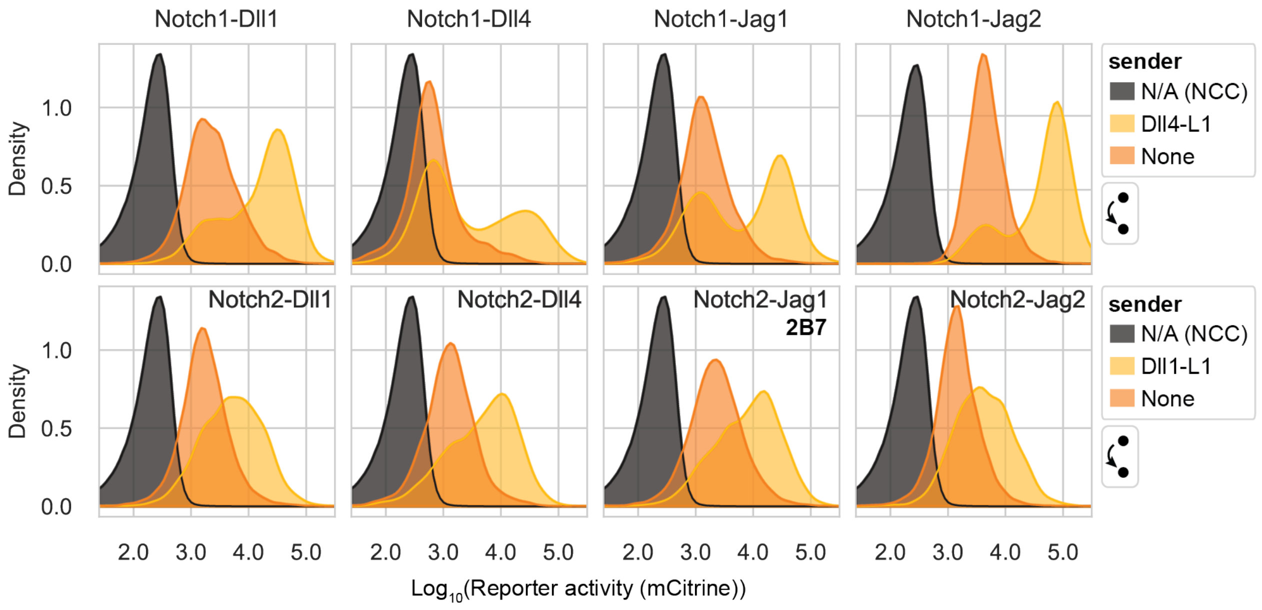


**Figure 5—figure supplement 4: CHO-K1 receiver clones' reporter dynamic ranges with minimum cis-ligand expression**

Histograms of reporter activity (mCitrine, A.U.) in the indicated receiver cells with minimum cis-ligand (maximum [4-epi-Tc]), transfected with Lfng and cultured with either wild-type CHO-K1 cells (sender = "None"; n = 7 biological replicates per receiver) or high-Dll1 or -Dll4 senders (for Notch2 and Notch1, respectively; n = 4 biological replicates per receiver). Each receiver histogram includes pooled data from all replicates. The averages of these distributions with CHO-K1 or Delta senders were used for background subtraction or y-axis normalization, respectively, in Figure 5A. Black histograms are CHO-K1 wild-type cells only (NCC = no color control).

bioRxiv preprint doi: https://doi.org/10.1101/2023.08.24.554677; this version posted August 25, 2023. The copyright holder for this preprint (which was not certified by peer review) is the author/funder, who has granted bioRxiv a license to display the preprint in perpetuity. It is made available under aCC-BY 4.0 International license.

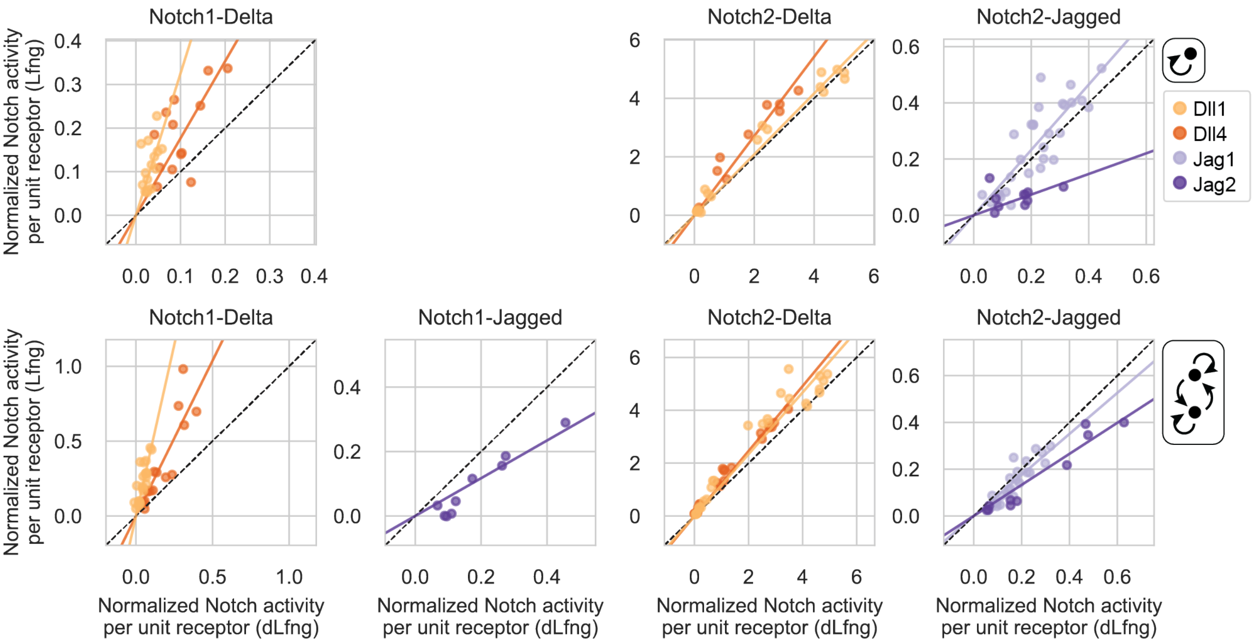


**Figure 5—figure supplement 5: Lfng effects on cis-activation and cis- + trans-activation**

Scatterplots comparing normalized signaling activities in receivers expressing Lfng (y-axis) vs. dLfng (x-axis) in the cis-activation assay (top row) and cis- + trans-activation assay (bottom row). Normalized signaling activities are identical to those in Figure 5A but include only the increasing phase for biphasic curves. Signaling activities below a signaling threshold of 0.05 were also excluded. Ligand-receptor combinations with fewer than five datapoints above the minimum signaling threshold were omitted. Each plot contains curves for one receptor and both cis-ligands of the same class (Delta or Jagged), and color indicates the cis-ligand for each curve. Solid lines are least-squares best-fits, and the black dashed line is y=x. See bootstrapped slope estimates in Figure 5C.

bioRxiv preprint doi: https://doi.org/10.1101/2023.08.24.554677; this version posted August 25, 2023. The copyright holder for this preprint (which was not certified by peer review) is the author/funder, who has granted bioRxiv a license to display the preprint in perpetuity. It is made available under aCC-BY 4.0 International license.

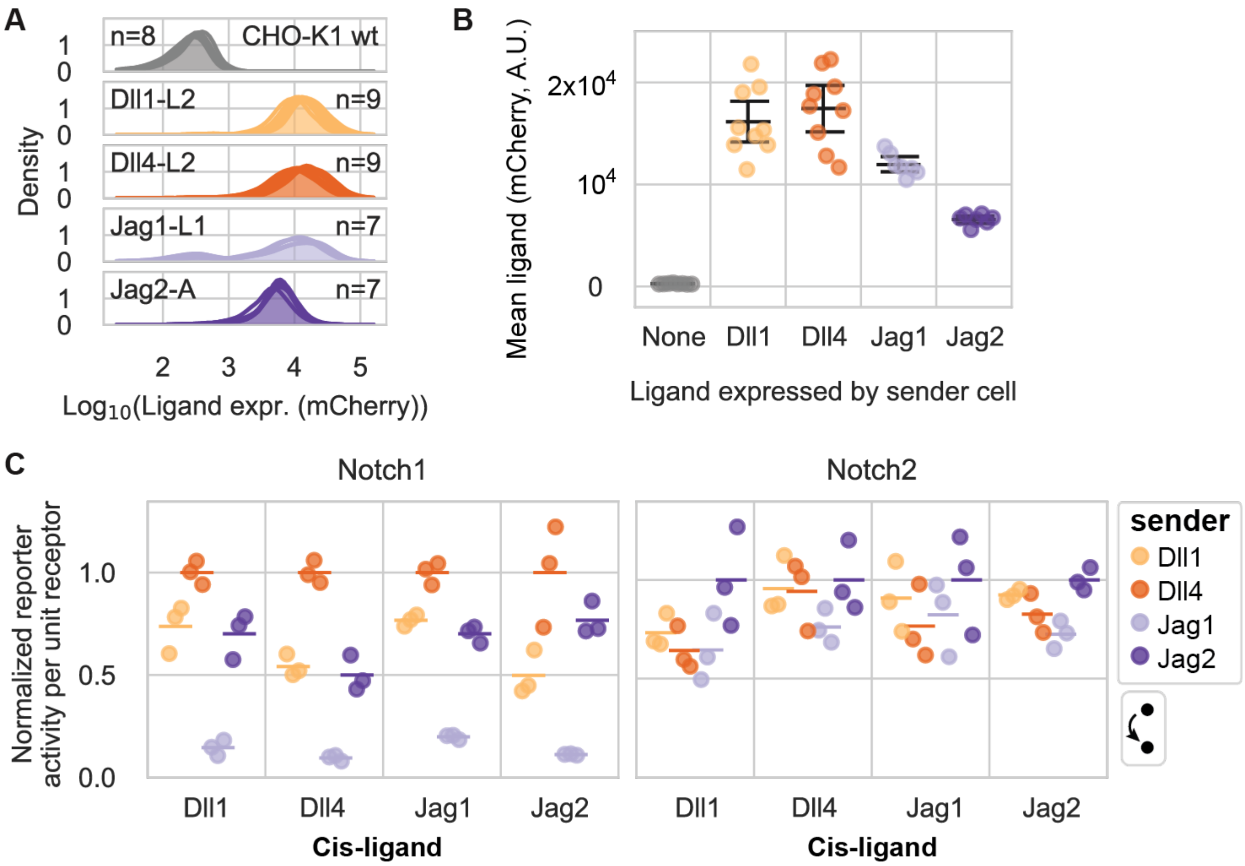


**Figure 6—figure supplement 1: Ligands' trans-activation strengths show greater diversity for Notch1 than Notch2**

(**A**) Single-cell distributions of cotranslational ligand expression (mCherry, A.U.) in the CHO-K1 sender populations used for this figure. Each histogram is a biological replicate, with a total of *n* replicates per plot.

(**B**) Mean cotranslational ligand expression (mCherry, A.U.) computed from histograms in (A). Black bars are the mean and 95% confidence intervals across 10,000 bootstrap replicates.

(**C**) Normalized signaling activity in receiver cells cultured with excess senders (A, B) in a trans-activation assay in the absence of cis-ligand expression (at maximum 4-epi-Tc). Cis-ligand is indicated on the x-axis, and colors denote the ligand expressed by the sender cells cultured in excess with a minority of receivers for a given datapoint. Normalized signaling activity is defined as reporter activity (mCitrine, A.U.) divided by cotranslational receptor expression (mTurq2, A.U.), normalized to the strongest signaling activity measured for each receiver clone in this experiment (average of three bioreplicates).

bioRxiv preprint doi: https://doi.org/10.1101/2023.08.24.554677; this version posted August 25, 2023. The copyright holder for this preprint (which was not certified by peer review) is the author/funder, who has granted bioRxiv a license to display the preprint in perpetuity. It is made available under aCC-BY 4.0 International license.

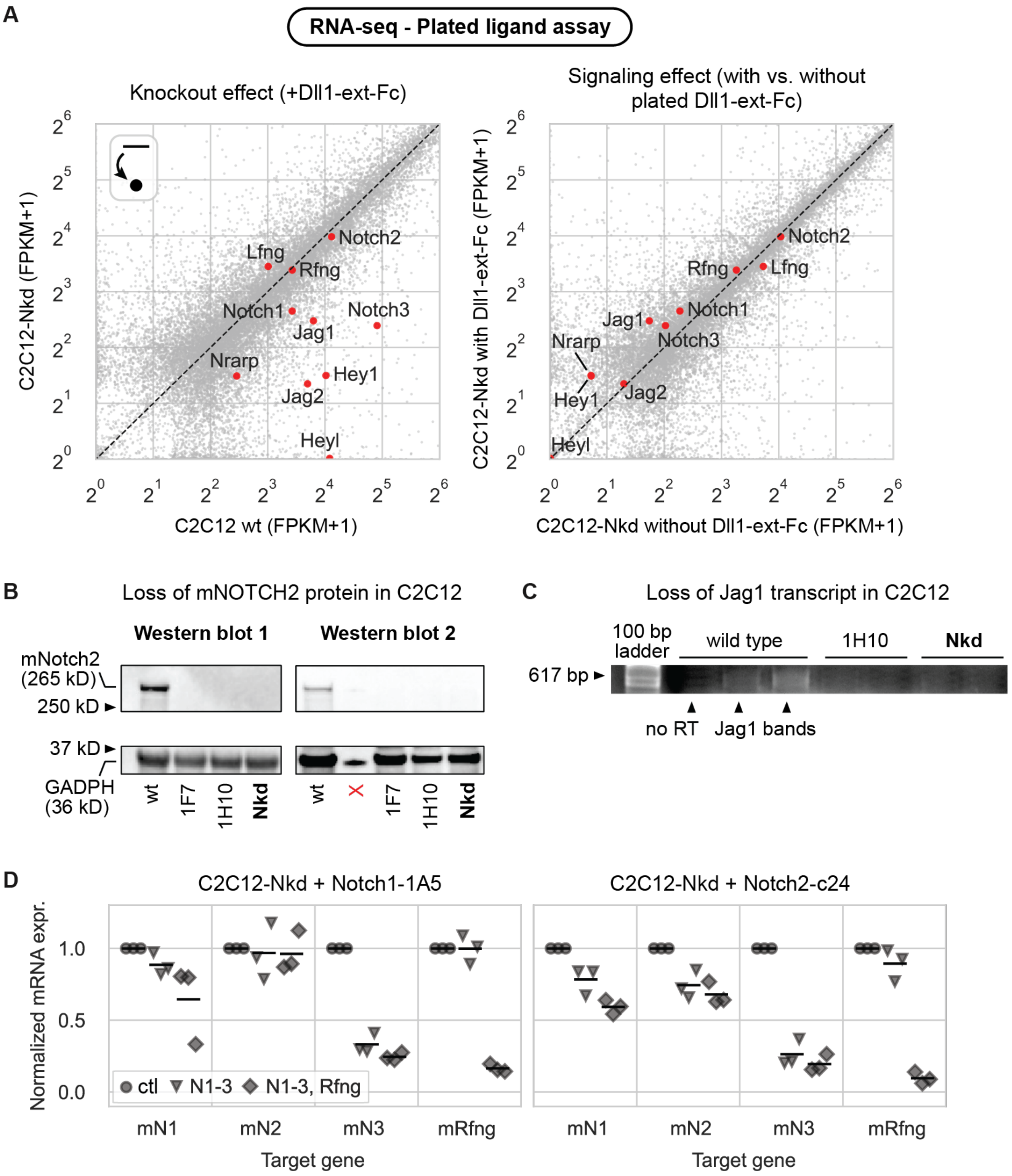


**Figure 7—figure supplement 1: CRISPR/Cas9 editing of C2C12 cells yields Notch-depleted clone C2C12-Nkd**

(**A**) Scatterplots of RNA transcript levels in RNA-seq data from wild-type C2C12 cells or the C2C12-Nkd ("Notch-depleted") clone generated in this study using CRISPR/Cas9 targeting deletion of mNotch2 and mJag1. Each data point is a transcript with a Fragments Per Kilobase of transcript per Million mapped reads (FPKM) value of ≥ 3 in at least one of the experimental

bioRxiv preprint doi: https://doi.org/10.1101/2023.08.24.554677; this version posted August 25, 2023. The copyright holder for this preprint (which was not certified by peer review) is the author/funder, who has granted bioRxiv a license to display the preprint in perpetuity. It is made available under aCC-BY 4.0 International license.

conditions. The left scatterplot shows differences in expression between wild-type cells and the C2C12-Nkd clone with Notch signaling stimulated by plated recombinant Dll1-ext-Fc in a plated ligand assay. The right scatterplot shows the effect of Notch ligand stimulation of C2C12-Nkd by comparing expression in the + vs. - plated Dll1-ext-Fc conditions. Transcripts corresponding to key Notch pathway and target genes are colored red and labeled.

(**B**) Western blot showing loss of endogenous Notch2 expression in CRISPR-treated C2C12 cell lines. (Left) First Western blot performed. (Right) Repeat Western blot to confirm the original results. The red X marks a well with no sample (signal is spillover from leftmost well).

(**C**) RT-PCR analysis of endogenous Jag1 transcript in CRISPR-treated C2C12 cell lines. Agarose gel electrophoresis of Jag1 RT-PCR product from C2C12 wt cells and 2 CRISPR treated cells lines, 1H10 and Nkd, shows loss of Jag1 transcript (617 bp band). No RT = “no reverse transcription” control.

(**D**) qRT-PCR of mouse Notch transcripts (labeled on x-axis) in Notch1 and Notch2 receiver cells derived from the C2C12-Nkd clone. Cells were treated with either a negative control siRNA, siRNAs against mNotch1, mNotch2, and mNotch3 (“N1-3”), or siRNAs against all three endogenous mouse receptors and mRfng (“N1-3, Rfng”). Y-axis values are $2^{-\Delta Cq}$ values (computed using mSdhA as a housekeeping gene) normalized to the expression level in the negative control knockdown for each gene within each bioreplicate. Black bars are the mean of three biological replicates.

bioRxiv preprint doi: https://doi.org/10.1101/2023.08.24.554677; this version posted August 25, 2023. The copyright holder for this preprint (which was not certified by peer review) is the author/funder, who has granted bioRxiv a license to display the preprint in perpetuity. It is made available under aCC-BY 4.0 International license.

## Tables

### Supplementary Table 1: Key resources

| Reagent type (species) or resource | Designation | Source or reference | Identifiers | Additional information |
|---|---|---|---|---|
| Gene (Homo sapiens) | Notch1 | NCBI ID: 4851 | | |
| Gene (Homo sapiens) | Notch2 | NCBI ID: 4853 | | |
| Gene (Homo sapiens) | Dll1 | NCBI ID: 28514 | | |
| Gene (Homo sapiens) | Dll4 | NCBI ID: 54567 | | |
| Gene (Homo sapiens) | Jag1 | NCBI ID: 182 | | |
| Gene (Homo sapiens) | Jag2 | NCBI ID: 3714 | | |
| Gene (Homo sapiens) | NGFR | NCBI ID: 4804 | | |
| Gene (Mus musculus) | Rfng | NCBI ID: 19719 | | |
| Gene (Mus musculus) | Lfng | NCBI ID: 16848 | | |
| Cell line (Cricetulus griseus) | CHO-K1 | ATCC | Cat# CCL-61 | |
| Cell line (Cricetulus griseus) | CHO Dll1 sender L1 | Derived from CHO-K1 | CHO-K1+CBh-Dll1-2xFLAG- T2A-H2B-mCherry - Sort level 1 (high mCherry) | |
| Cell line (Cricetulus griseus) | CHO Dll1 sender L4B | Derived from CHO-K1 | CHO-K1+CBh-Dll1-2xFLAG- T2A-H2B-mCherry - Sort level 4B (low mCherry) | |
| Cell line (Cricetulus griseus) | CHO Dll1 sender L4A | Derived from CHO-K1 | CHO-K1+CBh-Dll1-2xFLAG- T2A-H2B-mCherry - Sort level 4A (low mCherry) | |

bioRxiv preprint doi: https://doi.org/10.1101/2023.08.24.554677; this version posted August 25, 2023. The copyright holder for this preprint (which was not certified by peer review) is the author/funder, who has granted bioRxiv a license to display the preprint in perpetuity. It is made available under aCC-BY 4.0 International license.

| | | | | |
|---|---|---|---|---|
| Cell line (Cricetulus griseus) | CHO Dll1 sender L4 | Derived from CHO-K1 | CHO-K1+CBh-Dll1-2xFLAG- T2A-H2B-mCherry - Sort level 4 (medium mCherry) | |
| Cell line (Cricetulus griseus) | CHO Dll1 sender L2 | Derived from CHO-K1 | CHO-K1+CBh-Dll1-2xFLAG- T2A-H2B-mCherry - Sort level L2 (high mCherry) | |
| Cell line (Cricetulus griseus) | CHO Dll4 sender L1 | Derived from CHO-K1 | CHO-K1+CBh-Dll4-2xFLAG- T2A-H2B-mCherry - Sort level 1 (high mCherry) | |
| Cell line (Cricetulus griseus) | CHO Dll4 sender L4B | Derived from CHO-K1 | CHO-K1+CBh-Dll4-2xFLAG- T2A-H2B-mCherry - Sort level 4B (medium mCherry) | |
| Cell line (Cricetulus griseus) | CHO Dll4 sender L4 | Derived from CHO-K1 | CHO-K1+CBh-Dll4-2xFLAG- T2A-H2B-mCherry - Sort level 4 (medium mCherry) | |
| Cell line (Cricetulus griseus) | CHO Dll4 sender L2 | Derived from CHO-K1 | CHO-K1+CBh-Dll4-2xFLAG- T2A-H2B-mCherry - Sort level 2 (medium mCherry) | |
| Cell line (Cricetulus griseus) | CHO Jag1 sender L1 | Derived from CHO-K1 | CHO-K1+CBh-Jag1-2xFLAG-T2A-H2B-mCherry - Sort level 1 (high mCherry) | |
| Cell line (Cricetulus griseus) | CHO Jag1 sender L2 | Derived from CHO-K1 | CHO-K1+CBh-Jag1-2xFLAG-T2A-H2B-mCherry - Sort level 2 (medium mCherry) | |
| Cell line (Cricetulus griseus) | CHO Jag1 sender L3 | Derived from CHO-K1 | CHO-K1+CBh-Jag1-2xFLAG-T2A-H2B-mCherry - Sort level 3 (low mCherry) | |
| Cell line (Cricetulus griseus) | CHO Jag2 sender A | Derived from CHO-K1 | CHO-K1+CBh-Jag2-2xFLAG-T2A-H2B-mCherry - Sort level A (high mCherry) | |
| Cell line (Cricetulus griseus) | CHO Jag2 sender AB | Derived from CHO-K1 | CHO-K1+CBh-Jag2-2xFLAG-T2A-H2B-mCherry - Sort level AB (medium mCherry) | |
| Cell line (Cricetulus griseus) | CHO Jag2 sender AA | Derived from CHO-K1 | CHO-K1+CBh-Jag2-2xFLAG-T2A-H2B-mCherry - Sort level AA (high mCherry) | |
| Cell line (Cricetulus griseus) | CHO Jag2 sender B | Derived from CHO-K1 | CHO-K1+CBh-Jag2-2xFLAG-T2A-H2B-mCherry - Sort level B (low mCherry) | |

bioRxiv preprint doi: https://doi.org/10.1101/2023.08.24.554677; this version posted August 25, 2023. The copyright holder for this preprint (which was not certified by peer review) is the author/funder, who has granted bioRxiv a license to display the preprint in perpetuity. It is made available under aCC-BY 4.0 International license.

| | | | | |
|---|---|---|---|---|
| Cell line (Cricetulus griseus) | CHO Tet-OFF Dll1 sender G1 | Derived from CHO-K1 | CHO-K1+Tet-OFF Dll1-2xFLAG-T2A-H2B- mCherry G1 | |
| Cell line (Cricetulus griseus) | CHO Tet-OFF Dll4 sender 2D6 | Derived from CHO-K1 | CHO-K1+Tet-OFF Dll4-2xFLAG-T2A-H2B- mCherry 2D6 | |
| Cell line (Cricetulus griseus) | CHO Tet-OFF Jag1 sender 1G3 | Derived from CHO-K1 | CHO-K1+Tet-OFF Jag1-2xFLAG-T2A-H2B- mCherry 1G3 | |
| Cell line (Cricetulus griseus) | CHO Tet-OFF Jag2 sender 1H6 | Derived from CHO-K1 | CHO-K1+Tet-OFF Jag2-2xFLAG-T2A-H2B- mCherry 1H6 | |
| Cell line (Cricetulus griseus) | CHO reporter | Derived from CHO-K1 | CHO-K1+ 2xHS4-UAS-H2B-Citrine-<br>2xHS4 H1 | |
| Cell line (Cricetulus griseus) | CHO N1 receiver 2D1 | Derived from CHO reporter | CHO-K1+ 2xHS4-UAS-H2B-Citrine-<br>2xHS4+ PB-PGK-N1ECD-Gal4esn- T2A-H2B-mTurq2 2-D1 | |
| Cell line (Cricetulus griseus) | CHO N2 receiver 2A4 | Derived from CHO reporter | CHO-K1+ 2xHS4-UAS-H2B-Citrine-<br>2xHS4+ PB-PGK-N2ECD-Gal4esn- T2A-H2B-mTurq2 2-A4 | |
| Cell line (Cricetulus griseus | CHO N1-Tet-OFF Dll1 2G5 | Derived from CHO reporter | CHO-K1+ 2xHS4-UAS-H2B-Citrine- 2xHS4+Tet-OFF Dll1-2xFLAG-T2A-H2B- mCherry+ PB-CAG-N1ECD-Gal4esn- T2A-H2B-mTurq2 2-G5 | |
| Cell line (Cricetulus griseus | CHO N1-Tet-OFF Dll4 1G11 | Derived from CHO reporter | CHO-K1+ 2xHS4-UAS-H2B-Citrine- 2xHS4+Tet-OFF Dll4-2xFLAG-T2A-H2B- mCherry+ PB-CAG-N1ECD-Gal4esn- T2A-H2B-mTurq2 1-G11 | |
| Cell line (Cricetulus griseus | CHO N1-Tet-OFF Jag1 1E2 | Derived from CHO reporter | CHO-K1+ 2xHS4-UAS-H2B-Citrine- 2xHS4+Tet-OFF Jag1-2xFLAG-T2A-H2B- mCherry+ PB-CAG-N1ECD-Gal4esn-T2A-H2B-mTurq2 1-E2 | |

bioRxiv preprint doi: https://doi.org/10.1101/2023.08.24.554677; this version posted August 25, 2023. The copyright holder for this preprint (which was not certified by peer review) is the author/funder, who has granted bioRxiv a license to display the preprint in perpetuity. It is made available under aCC-BY 4.0 International license.

| | | | | |
|---|---|---|---|---|
| Cell line (Cricetulus griseus | CHO N1-Tet-OFF Jag2 2E10 | Derived from CHO reporter | CHO-K1+ 2xHS4-UAS-H2B-Citrine- 2xHS4+Tet-OFF Jag2-2xFLAG-T2A-H2B- mCherry+ PB-CAG-N1ECD-Gal4esn- T2A-H2B-mTurq2 2-E10 | |
| Cell line (Cricetulus griseus | CHO N2-Tet-OFF Dll1 1C4 | Derived from CHO reporter | CHO-K1+ 2xHS4-UAS-H2B-Citrine- 2xHS4+Tet-OFF Dll1-2xFLAG-T2A-H2B- mCherry+ PB-CAG-N2ECD-Gal4esn- T2A-H2B-mTurq2 1-C4 | |
| Cell line (Cricetulus griseus | CHO N2-Tet-OFF Dll4 1B8 | Derived from CHO reporter | CHO-K1+ 2xHS4-UAS-H2B-Citrine- 2xHS4+Tet-OFF Dll4-2xFLAG-T2A-H2B- mCherry+ PB-CAG-N2ECD-Gal4esn- T2A-H2B-mTurq2 1-B8 | |
| Cell line (Cricetulus griseus | CHO N2-Tet-OFF Jag1 2B8 | Derived from CHO reporter | CHO-K1+ 2xHS4-UAS-H2B-Citrine- 2xHS4+Tet-OFF Jag1-2xFLAG-T2A-H2B- mCherry+ PB-CAG-N2ECD-Gal4esn- T2A-H2B-mTurq2 2-B8 | |
| Cell line (Cricetulus griseus | CHO N2-Tet-OFF Jag1 2B7 | Derived from CHO reporter | CHO-K1+ 2xHS4-UAS-H2B-Citrine- 2xHS4+Tet-OFF Jag1-2xFLAG-T2A-H2B- mCherry+ PB-CAG-N2ECD-Gal4esn- T2A-H2B-mTurq2 2-B7 | |
| Cell line (Cricetulus griseus | CHO N2-Tet-OFF Jag2 1A1 | Derived from CHO reporter | CHO-K1+ 2xHS4-UAS-H2B-Citrine- 2xHS4+Tet-OFF Jag2-2xFLAG-T2A-H2B- mCherry+ PB-CAG-N2ECD-Gal4esn- T2A-H2B-mTurq2 1-A1 | |
| Cell line (Cricetulus griseus | CHO N1-Tet-OFF H2B-mCherry | Derived from CHO reporter | CHO-K1+ 2xHS4-UAS-H2B-Citrine- 2xHS4+Tet-OFF H2B-mCherry+ PB-CAG-N1ECD-Gal4esn- T2A-H2B-mTurq2 F4 | |
| Cell line (Cricetulus griseus | CHO N2-Tet-OFF H2B-mCherry | Derived from CHO reporter | CHO-K1+ 2xHS4-UAS-H2B-Citrine- 2xHS4+Tet-OFF H2B-mCherry+ PB-CAG-N2ECD-Gal4esn- T2A-H2B-mTurq2 G2 | |
| Cell line (Cricetulus griseus | CHO N1-Tet-OFF NGFR-T2A- H2B-mCherry | Derived from CHO reporter | CHO-K1+ 2xHS4-UAS-H2B-Citrine- 2xHS4+Tet-OFF NGFR-T2A-H2B-mCherry+ PB-CAG-N1ECD-Gal4esn- T2A-H2B-mTurq2 2-A6 | |

bioRxiv preprint doi: https://doi.org/10.1101/2023.08.24.554677; this version posted August 25, 2023. The copyright holder for this preprint (which was not certified by peer review) is the author/funder, who has granted bioRxiv a license to display the preprint in perpetuity. It is made available under aCC-BY 4.0 International license.

| | | | | |
|---|---|---|---|---|
| Cell line (Cricetulus griseus | CHO N2-Tet-OFF NGFR-T2A- H2B-mCherry | Derived from CHO reporter | CHO-K1+ 2xHS4-UAS-H2B-Citrine- 2xHS4+Tet-OFF NGFR-T2A-H2B-mCherry+ PB-CAG-N2ECD-Gal4esn- T2A-H2B-mTurq2 1-H4 | |
| Cell line (*Mus musculus*) | C2C12 | Cat# CRL-1772 | ATCC | |
| Cell line (*Mus musculus*) | C2C12-Nkd | Derived from C2C12 | C2C12 with N2 and Jag1 CRISPR knockout | |
| Cell line (*Mus musculus*) | C2C12 reporter | Derived from C2C12-Nkd | C2C12-Nkd + 2xHS4-UAS-H2B-Citrine- 2xHS4 B5 | |
| Cell line (*Mus musculus*) | C2C12 N1 receiver | Derived from C2C12 reporter | C2C12-Nkd + 2xHS4-UAS-H2B-Citrine -2xHS4+ PB-CAG-N1ECD-Gal4esn- T2A-H2B-mTurq2 1-A5 | |
| Cell line (*Mus musculus*) | C2C12 N2 receiver | Derived from C2C12 reporter | C2C12-Nkd + 2xHS4-UAS-H2B-Citrine -2xHS4+ PB-CAG-N2ECD-Gal4esn- T2A-H2B-mTurq2 c24 | |
| Cell line (*Mus musculus*) | C2C12 Dll1 sender c19 | Derived from C2C12-Nkd | C2C12-Nkd+ CBh-Dll1-2xFLAG-T2A- H2B-mCherry c19 (high mCherry) | |
| Cell line (*Mus musculus*) | C2C12 Dll4 sender 2-H10 | Derived from C2C12-Nkd | C2C12-Nkd+ CBh-Dll4-2xFLAG-T2A- H2B-mCherry 2-H10 (high mCherry) | |
| Cell line (*Mus musculus*) | C2C12 Jag1 sender c11 | Derived from C2C12-Nkd | C2C12-Nkd+ CBh-Jag1-2xFLAG-T2A- H2B-mCherry c11 (high mCherry) | |
| Cell line (*Mus musculus*) | C2C12 N1-Tet-OFF NGFR | Derived from C2C12 reporter | C2C12+ 2xHS4-UAS-H2B-Citrine- 2xHS4+Tet-OFF NGFR-2xFLAG-T2A-H2B- mCherry+ PB-CAG-N1ECD-Gal4esn- T2A-H2B-mTurq2 1-H6 | |
| Cell line (*Mus musculus*) | C2C12 N2-Tet-OFF NGFR | Derived from C2C12 reporter | C2C12+ 2xHS4-UAS-H2B-Citrine- 2xHS4+Tet-OFF NGFR-2xFLAG-T2A-H2B- mCherry+ PB-CAG-N2ECD-Gal4esn- T2A-H2B-mTurq2 1-E9 | |

bioRxiv preprint doi: https://doi.org/10.1101/2023.08.24.554677; this version posted August 25, 2023. The copyright holder for this preprint (which was not certified by peer review) is the author/funder, who has granted bioRxiv a license to display the preprint in perpetuity. It is made available under aCC-BY 4.0 International license.

| | | | | |
|---|---|---|---|---|
| Cell line (*Mus musculus*) | C2C12 N1-Tet-OFF Dll4 | Derived from C2C12 reporter | C2C12+ 2xHS4-UAS-H2B-Citrine- 2xHS4+Tet-OFF Dll4-2xFLAG-T2A-H2B- mCherry+ PB-CAG-N1ECD-Gal4esn- T2A-H2B-mTurq2 2-D1 | |
| Cell line (*Mus musculus*) | C2C12 N2-Tet-OFF Dll4 | Derived from C2C12 reporter | C2C12+ 2xHS4-UAS-H2B-Citrine- 2xHS4+Tet-OFF Dll4-2xFLAG-T2A-H2B- mCherry+ PB-CAG-N2ECD-Gal4esn- T2A-H2B-mTurq2 3-B12 | |
| Cell line (*Mus musculus*) | C2C12 N1-Tet-OFF Jag2 | Derived from C2C12 reporter | C2C12+ 2xHS4-UAS-H2B-Citrine- 2xHS4+Tet-OFF Jag2-2xFLAG-T2A-H2B- mCherry+ PB-CAG-N1ECD-Gal4esn- T2A-H2B-mTurq2 1-E7 | |
| Cell line (*Mus musculus*) | C2C12 N2-Tet-OFF Jag2 | Derived from C2C12 reporter | C2C12+ 2xHS4-UAS-H2B-Citrine- 2xHS4+Tet-OFF Jag2-2xFLAG-T2A-H2B- mCherry+ PB-CAG-N2ECD-Gal4esn- T2A-H2B-mTurq2 1-B12 | |
| Transfected construct (recombinant DNA) | Super PiggyBac Transposase Expression Vector | System Biosciences | Cat# PB210PA-1 | Transposase used in all transfections with PiggyBac vectors |
| Transfected construct (recombinant DNA) | pEV-2xHS4-UAS-H2B-Citrine-2xHS4 | This paper | | Reporter for hNotch-Gal4 receptors in cell lines |
| Transfected construct (recombinant DNA) | PB-CMV-MCS-EF1-Puro (PiggyBac vector) | System Biosciences | Cat# PB510B-1 | Base vector used to derive all PiggyBac constructs |
| Transfected construct (recombinant DNA) | PB-PGK-N1ECD-Gal4esn-T2A-H2B-mTurq2 | This paper | | Notch1ECD-Gal4 synthetic receptor in CHO receiver cells |
| Transfected construct (recombinant DNA) | PB-PGK-N2ECD-Gal4esn-T2A-H2B-mTurq2 | This paper | | Notch2ECD-Gal4 synthetic receptor in CHO receiver cells |

bioRxiv preprint doi: https://doi.org/10.1101/2023.08.24.554677; this version posted August 25, 2023. The copyright holder for this preprint (which was not certified by peer review) is the author/funder, who has granted bioRxiv a license to display the preprint in perpetuity. It is made available under aCC-BY 4.0 International license.

| | | | | |
|---|---|---|---|---|
| Transfected construct (recombinant DNA) | PB-CAG-N1ECD-Gal4esn-T2A-H2B-mTurq2 | This paper | | Notch1ECD-Gal4 synthetic receptor in Tet-OFF cell lines and C2C12 receiver cells |
| Transfected construct (recombinant DNA) | PB-CAG-N2ECD-Gal4esn-T2A-H2B-mTurq2 | This paper | | Notch2ECD-Gal4 synthetic receptor in Tet-OFF cell lines and C2C12 receiver cells |
| Transfected construct (recombinant DNA) | pCW57.1-MAT2A | Addgene | Cat# 100521 | Base vector used to derive all Tet-OFF constructs and constitutive ligand constructs |
| Transfected construct (recombinant DNA) | pCW57.1 Tet-OFF Dll1-2xFLAG-T2A-H2B- mCherry | This paper | | Repressible Dll1 ligand in cell lines. Used to make lentivirus. |
| Transfected construct (recombinant DNA) | pCW57.1 Tet-OFF Dll4-2xFLAG-T2A-H2B- mCherry | This paper | | Repressible Dll4 ligand in cell lines. Used to make lentivirus. |
| Transfected construct (recombinant DNA) | pCW57.1 Tet-OFF Jag1-2xFLAG-T2A-H2B- mCherry | This paper | | Repressible Jag1 ligand in cell lines. Used to make lentivirus. |
| Transfected construct (recombinant DNA) | pCW57.1 Tet-OFF Jag2-2xFLAG-T2A-H2B- mCherry | This paper | | Repressible Jag2 ligand in cell lines. Used to make lentivirus. |
| Transfected construct (recombinant DNA) | pCW57.1 Tet-OFF H2B-mCherry | This paper | | Repressible H2B-mCherry used as negative control in cell lines. Used to make lentivirus. |

bioRxiv preprint doi: https://doi.org/10.1101/2023.08.24.554677; this version posted August 25, 2023. The copyright holder for this preprint (which was not certified by peer review) is the author/funder, who has granted bioRxiv a license to display the preprint in perpetuity. It is made available under aCC-BY 4.0 International license.

| | | | | |
|---|---|---|---|---|
| Transfected construct (recombinant DNA) | pCW57.1 Tet-OFF NGFR-T2A-H2B-mCherry | This paper | | Repressible NGFR ligand used as negative control in cell lines. Used to make lentivirus. |
| Transfected construct (recombinant DNA) | pCW57.1 CBh-Dll1-2xFLAG-T2A-H2B-mCherry | This paper | | Constitutively expressed Dll1 ligand in cell lines. Used to make lentivirus. |
| Transfected construct (recombinant DNA) | pCW57.1 CBh-Dll4-2xFLAG-T2A-H2B-mCherry | This paper | | Constitutively expressed Dll4 ligand in cell lines. Used to make lentivirus. |
| Transfected construct (recombinant DNA) | pCW57.1 CBh-Jag1-2xFLAG-T2A-H2B-mCherry | This paper | | Constitutively expressed Jag1 ligand in cell lines. Used to make lentivirus. |
| Transfected construct (recombinant DNA) | pCW57.1 CBh-Jag2-2xFLAG-T2A-H2B-mCherry | This paper | | Constitutively expressed Jag2 ligand in cell lines. Used to make lentivirus. |
| Transfected construct (recombinant DNA) | PB-CMV-Lfng-BGHpA-EF1-Puro | Lebon et al. 2014 | | Constitutively expressed Lfng in cell lines treated with siRNA |
| Transfected construct (recombinant DNA) | PB-CMV-Lfng(D289E)-BGHpA-EF1-Puro | This paper | | Constitutively expressed dLfng (D289E) in cell lines treated with siRNA. Sequence from (Luther et al., 2009) |

bioRxiv preprint doi: https://doi.org/10.1101/2023.08.24.554677; this version posted August 25, 2023. The copyright holder for this preprint (which was not certified by peer review) is the author/funder, who has granted bioRxiv a license to display the preprint in perpetuity. It is made available under aCC-BY 4.0 International license.

| | | | | |
|---|---|---|---|---|
| Transfected construct (recombinant DNA) | PB-CMV7-IFP2.0-BGHpA-SV40-Neo | | | Vector co-transfected with fringe plasmids |
| Transfected construct (recombinant DNA) | pX330 (CRISPR-Cas9 plasmid system) | Nandagopal et al. 2019 Cong et al. 2013 | | Plasmid used to insert RNA guide sequences for use in CRISPR knockdown in C2C12 cells |
| Antibody | PE anti-human Notch1 antibody MHN1-519 | BioLegend | Cat# 352105 | Used in soluble ligand binding assay |
| Antibody | PE anti-human Notch2 antibody MHN2-25 | BioLegend | Cat# 348303 | Used in soluble ligand binding assay |
| Antibody | goat anti-human IgG Alexa Fluor 594 | Thermo Fisher Scientific | Cat# A-11014 | Secondary antibody used to precluster ligands in soluble ligand binding assay |
| Antibody | Rabbit anti-Notch2 | Cell Signaling Technology | Cat# 5732 | For Westerns |
| Antibody | Rabbit anti-GAPDH | Cell Signaling Technology | Cat# 2118 | For Westerns |
| Antibody | Amersham ECL Rabbit IgG, HRP-linked whole Ab | Cytiva | Cat# NA934 | For Westerns |
| siRNA | AllStars Neg. Control siRNA | Qiagen | Cat# 1027281 | |
| siRNA | Custom Select siRNA hamster Rfng | Thermo Fisher Scientific | Cat# 4399666 ID# s553138 | Sequence (5' to 3') GCUGUAAAAU GUCAGUGGAtt |
| siRNA | Custom Select siRNA hamster Lfng | Thermo Fisher Scientific | Cat# 4399665 ID# 555728 | Sequence (5' to 3') AGCUAAUGAU GAUAAGGGAtt |

bioRxiv preprint doi: https://doi.org/10.1101/2023.08.24.554677; this version posted August 25, 2023. The copyright holder for this preprint (which was not certified by peer review) is the author/funder, who has granted bioRxiv a license to display the preprint in perpetuity. It is made available under aCC-BY 4.0 International license.

| | | | | |
|---|---|---|---|---|
| siRNA | Silencer Select siRNA mouse N1 | Thermo Fisher Scientific | Cat # 4390771<br>ID# s70699 | |
| siRNA | Custom Stealth siRNA mouse N2 | Thermo Fisher Scientific | Cat # 10620312<br>ID# 359602D12 | Sequence (5' to 3') GACCUUCACC CAUCCUGCAA GUUCA |
| siRNA | Stealth siRNA mouse N3 | Thermo Fisher Scientific | Cat# 1320001<br>ID# mss207111 | |
| siRNA | Silencer Select siRNA mouse Rfng | Thermo Fisher Scientific | Cat# 4390771<br>ID# s72908 | |
| Reagent | Zeocin | Thermo Fisher Scientific | Cat# R25001 | |
| Reagent | Geneticin | Thermo Fisher Scientific | Cat# 10131-035 | |
| Reagent | Blasticidin | Invivogen | Cat# ANT-BL-1 | |
| Reagent | HBSS, no calcium, no magnesium, no phenol red | Thermo Fisher Scientific | Cat# 14175-095 | |
| Reagent | Alpha MEM Earle's Salts | FUJIFILM Irvine Scientifc | Cat #9144-500 | |
| Reagent | DMEM high glucose, no glutamine | Thermo Fisher Scientific | Cat# 11960069 | |
| Reagent | Fetal Bovine Serum (FBS) | Avantor VWR | Cat# 97068-085<br>Lot# 323K20 | |
| Reagent | 100X Sodium Pyruvate | Thermo Fisher Scientific | Cat# 11360-070 | |
| Reagent | 100X Penicillin, Streptomysin, Glutamine | Thermo Fisher Scientific | Cat# 10378-016 | |
| Reagent | Lipfectamine RNAiMAX Transfection Reagent | Thermo Fisher Scientific | Cat# 13778075 | |
| Reagent | Trypsin (0.25%), phenol red | Thermo Fisher Scientific | Cat# 15050065 | No EDTA |

bioRxiv preprint doi: https://doi.org/10.1101/2023.08.24.554677; this version posted August 25, 2023. The copyright holder for this preprint (which was not certified by peer review) is the author/funder, who has granted bioRxiv a license to display the preprint in perpetuity. It is made available under aCC-BY 4.0 International license.

| | | | | |
|---|---|---|---|---|
| Reagent | Polyplus-transfection jetOPTIMUS DNA Transfection Reagent | Genesee Scientific | Cat# 55-250 | |
| Protein | Bovine Serum Albumin | Sigma Aldrich | Cat# A4503 | |
| Protein | Cas9 Protein | PNA Bio Inc | Cat# CP01 | |
| Peptide, recombinant protein | Recombinant Human Dll1ext-Fc fusion proteins | (Sprinzak et al., 2010) | Kindly provided by Irwin Bernstein, MD at Fred Hutchinson Cancer Reserch Center | |
| Peptide, recombinant protein | Recombinant Human IgG1 Fc Protein | Bio-Techne/R&D Systems | Cat# 110-HG-100 | |
| Peptide, recombinant protein | Recombinant Human DLL1 Fc Chimera Protein | Bio-Techne/R&D Systems | Cat# 10184-DL-050 | |
| Peptide, recombinant protein | Recombinant Human DLL4 Fc Chimera Protein | Bio-Techne/R&D Systems | Cat# 10185-D4-050 | |
| Peptide, recombinant protein | Recombinant Human Jag1 Fc Chimera Protein | Bio-Techne/R&D Systems | Cat# 1277-JG-050 | |
| Peptide, recombinant protein | Recombinant Human Jag2 Fc Chimera Protein | Bio-Techne/R&D Systems | Cat# 1726-JG-050 | |
| Chemical compound, drug | DAPT | Sigma Aldrich | Cat# D5942 | |
| Chemical compound, drug | 4-epi tetracycline hydrochloride | Sigma Aldrich | Cat# 37918 | |
| Commercial assay or kit | Megashortscript T7 transcription kit | Thermo Fisher Scientific | Cat# AM1354 | |
| Commercial assay or kit | RNeasy Mini Kit | Qiagen | Cat# 74104 | |
| Commercial assay or kit | QIAshredder | Qiagen | Cat# 79656 | |
| Commercial assay or kit | iScript cDNA Sythesis Kit | Bio-Rad | Cat# 1708890 | |

bioRxiv preprint doi: https://doi.org/10.1101/2023.08.24.554677; this version posted August 25, 2023. The copyright holder for this preprint (which was not certified by peer review) is the author/funder, who has granted bioRxiv a license to display the preprint in perpetuity. It is made available under aCC-BY 4.0 International license.

| | | | | |
|---|---|---|---|---|
| Commercial assay or kit | MycoStrip | InvivoGen | Cat# rep-mys-100 | |
| CHO-K1 RNA-seq database | https://anksi.shinyapps.io/biosciences/ | | (Singh et al., 2018) | |
| Software, algorithm | RNA-sequencing read trimming and quality control | TrimGalore | https://www.bioinformatics.babraham.ac.uk/projects/trim_galore/ | |
| Software, algorithm | RNA-sequencing read alignment | HISAT2 v2.1.0 (via Galaxy) | http://daehwankimlab.github.io/hisat2/; https://usegalaxy.org/ | |
| Software, algorithm | RNA-sequencing transcript abundance calculation | StringTie v1.3.4 (via Galaxy) | http://ccb.jhu.edu/software/stringtie/; https://usegalaxy.org/ | |
| Software, algorithm | Import of flow cytometry .fcs files for data processing in Python3 | FlowCal | https://flowcal.readthedocs.io/en/latest/; (Castillo-Hair et al., 2016) | |

bioRxiv preprint doi: https://doi.org/10.1101/2023.08.24.554677; this version posted August 25, 2023. The copyright holder for this preprint (which was not certified by peer review) is the author/funder, who has granted bioRxiv a license to display the preprint in perpetuity. It is made available under aCC-BY 4.0 International license.

**Supplementary Table 2: Notch gene expression in wild-type CHO-K1 cells**

| Gene | Expression (CPM) |
|---|---|
| Notch1 | 6.66 |
| Notch2 | 192.67 |
| Notch3 | 8.14 |
| Notch4 | 0.43 |
| Jag1 | 9.58 |
| Lfng | 3.97 |
| Rfng | 71.51 |

These expression values in counts per million reads mapped (CPM) were obtained from the CHO gene expression visualization application (CGEVA) database of CHO RNA-seq data, including from wild-type CHO-K1 cells, located at https://anksi.shinyapps.io/biosciences/ (Singh et al., 2018). Dll1, Dll4, Jag2, and Mfng were not available in the database. See also Methods.

bioRxiv preprint doi: https://doi.org/10.1101/2023.08.24.554677; this version posted August 25, 2023. The copyright holder for this preprint (which was not certified by peer review) is the author/funder, who has granted bioRxiv a license to display the preprint in perpetuity. It is made available under aCC-BY 4.0 International license.

**Supplementary Table 3: 4-epi-Tc concentrations used to induce ligand expression**

| Experiment | Cell type | Cell line (clone) | [4-epi-Tc] (ng/mL) |
|---|---|---|---|
| Figure 4B; Figure 1-S1 | CHO-K1 | Notch1-H2B-mCh (F4) | 0, 5, 10, 15, 20, 50, 500 |
| Figure 4B; Figure 1-S1 | CHO-K1 | Notch1-NGFR (2A6) | 0, 5, 10, 15, 20, 50, 500 |
| Figure 4B; Figure 1-S1 | CHO-K1 | Notch1-Dll1 (2G5) | 0, 20, 50, 100, 200, 300, 500 |
| Figure 4B; Figure 1-S1 | CHO-K1 | Notch1-Dll4 (1G11) | 0, 40, 70, 100, 150, 200, 500 |
| Figure 4B; Figure 1-S1 | CHO-K1 | Notch1-Jag1 (1E2) | 0, 20, 50, 100, 200, 300, 500 |
| Figure 4B; Figure 1-S1 | CHO-K1 | Notch1-Jag2 (2E10) | 0, 40, 70, 100, 150, 200, 800 |
| Figure 4B; Figure 1-S1 | CHO-K1 | Notch2-H2B-mCh (G2) | 0, 5, 10, 15, 20, 50, 500 |
| Figure 4B; Figure 1-S1 | CHO-K1 | Notch2-NGFR (1H4) | 0, 5, 10, 15, 20, 50, 500 |
| Figure 4B; Figure 1-S1 | CHO-K1 | Notch2-Dll1 (1C4) | 0, 10, 20, 40, 100, 200, 500 |
| Figure 4B; Figure 1-S1 | CHO-K1 | Notch2-Dll4 (1B8) | 0, 10, 20, 40, 100, 200, 500 |
| Figure 4B; Figure 1-S1 | CHO-K1 | Notch2-Jag1 (2B8) | 0, 10, 20, 40, 100, 200, 500 |
| Figure 4B; Figure 1-S1 | CHO-K1 | Notch2-Jag2 (1A1) | 0, 10, 20, 40, 100, 200, 500 |
| Figure 4D | CHO-K1 | Notch2-Dll1 (1C4) | 10, 500 |
| Figure 4D | CHO-K1 | Notch2-Dll4 (1B8) | 10, 500 |
| Figure 6 | CHO-K1 | Notch1-Dll1 (2G5) | 0, 20, 50, 100, 200, 300, 500 |
| Figure 6 | CHO-K1 | Notch1-Dll4 (1G11) | 0, 40, 70, 100, 150, 200, 500 |
| Figure 6 | CHO-K1 | Notch1-Jag1 (1E2) | 0, 20, 50, 100, 200, 300, 500 |

bioRxiv preprint doi: https://doi.org/10.1101/2023.08.24.554677; this version posted August 25, 2023. The copyright holder for this preprint (which was not certified by peer review) is the author/funder, who has granted bioRxiv a license to display the preprint in perpetuity. It is made available under aCC-BY 4.0 International license.

| | | | |
|---|---|---|---|
| Figure 6 | CHO-K1 | Notch1-Jag2 (2E10) | 0, 40, 70, 100, 150, 200, 800 |
| Figure 6 | CHO-K1 | Notch2-Dll1 (1C4) | 0, 10, 20, 40, 100, 200, 500 |
| Figure 6 | CHO-K1 | Notch2-Dll4 (1B8) | 0, 10, 20, 40, 100, 200, 500 |
| Figure 6 | CHO-K1 | Notch2-Jag1 (2B8) | 0, 10, 20, 40, 100, 200, 500 |
| Figure 6 | CHO-K1 | Notch2-Jag2 (1A1) | 0, 10, 20, 40, 100, 200, 500 |
| Figure 7C | C2C12-Nkd | Notch1-NGFR (1H6) | 0, 10, 500 |
| Figure 7C | C2C12-Nkd | Notch1-Dll4 (2D1) | 0, 15, 500 |
| Figure 7C | C2C12-Nkd | Notch1-Jag2 (1E7) | 0, 25, 500 |
| Figure 7C | C2C12-Nkd | Notch2-NGFR (1E9) | 0, 10, 500 |
| Figure 7C | C2C12-Nkd | Notch2-Dll4 (3B12) | 0, 15, 500 |
| Figure 7C | C2C12-Nkd | Notch2-Jag2 (1B12) | 0, 25, 500 |
| Figure 1-S2B | CHO-K1 | Tet-OFF Dll1 (G1) | 10 |
| Figure 1-S2B | CHO-K1 | Tet-OFF Dll4 (2D6) | 5 |
| Figure 4-S2 | CHO-K1 | Tet-OFF Dll1 (G1) | 0 |
| Figure 4-S2 | CHO-K1 | Tet-OFF Dll4 (2D6) | 0 |
| Figure 1-S2A; Figure 2D; Figure 2-S2 | CHO-K1 | Tet-OFF Dll1 (G1) | 0, 20, 40, 60, 80, 100, 150, 500 |
| Figure 1-S2A; Figure 2D; Figure 2-S2 | CHO-K1 | Tet-OFF Dll4 (2D6) | 0, 10, 20, 30, 40, 60, 80, 500 |
| Figure 1-S2A; Figure 2D; Figure 2-S2 | CHO-K1 | Tet-OFF Jag1 (1G3) | 0, 5, 10, 15, 20, 35, 50, 500 |
| Figure 1-S2A; Figure 2D; Figure 2-S2 | CHO-K1 | Tet-OFF Jag2 (1H6) | 0, 5, 10, 15, 20, 25, 40, 500 |
| Figure 5A; Figure 5-S1; Figure 5-S2; Figure 5-S3; Figure 5-S5 | CHO-K1 | Notch1-Dll1 (2G5) | 0, 25, 50, 80, 500 |
| Figure 5A; Figure 5-S1; Figure 5-S2; Figure 5-S3; Figure 5-S5 | CHO-K1 | Notch1-Dll4 (1G11) | 0, 30, 50, 80, 120, 500 |

bioRxiv preprint doi: https://doi.org/10.1101/2023.08.24.554677; this version posted August 25, 2023. The copyright holder for this preprint (which was not certified by peer review) is the author/funder, who has granted bioRxiv a license to display the preprint in perpetuity. It is made available under aCC-BY 4.0 International license.

| | | | |
|---|---|---|---|
| Figure 5A; Figure 5-S1; Figure 5-S2 | CHO-K1 | Notch1-Jag1 (1E2) | 0, 5, 10, 20, 30, 500 |
| Figure 5A; Figure 5-S1; Figure 5-S2; Figure 5-S3; Figure 5-S5 | CHO-K1 | Notch1-Jag2 (2E10) | 0, 10, 20, 40, 800 |
| Figure 5A; Figure 5-S1; Figure 5-S2; Figure 5-S3; Figure 5-S5 | CHO-K1 | Notch2-Dll1 (1C4) | 0, 5, 15, 30, 500 |
| Figure 5A; Figure 5-S1; Figure 5-S2; Figure 5-S3; Figure 5-S5 | CHO-K1 | Notch2-Dll4 (1B8) | 0, 5, 15, 30, 500 |
| Figure 5A; Figure 5-S1; Figure 5-S2; Figure 5-S3; Figure 5-S5 | CHO-K1 | Notch2-Jag1 (2B7) | 0, 5, 15, 30, 500 |
| Figure 5A; Figure 5-S1; Figure 5-S2; Figure 5-S3; Figure 5-S5 | CHO-K1 | Notch2-Jag2 (1A1) | 0, 10, 25, 50, 500 |

*Where Tet-OFF inducible sender or receiver cells were used but don't appear in the table above, 4-epi-Tc was used at a concentration of 500-800 ng/mL to fully suppress expression.*

bioRxiv preprint doi: https://doi.org/10.1101/2023.08.24.554677; this version posted August 25, 2023. The copyright holder for this preprint (which was not certified by peer review) is the author/funder, who has granted bioRxiv a license to display the preprint in perpetuity. It is made available under aCC-BY 4.0 International license.

**Supplementary Table 4: Sender-receiver cell pairs used for coculture assays**

Where multiple receivers are listed, they were cultured with the same senders but in separate wells (receivers were not mixed together). All experiments with CHO-K1 cells used wild-type cells as negative sending controls and experiments with C2C12-Nkd receivers used the blank C2C12-Nkd parental line as negative controls. Clone identifiers are in parentheses; polyclonal cell lines lack parentheses. See senders' ligand expression distributions in Figure 1C, Figure 1—figure supplement 2A, and Figure 6—figure supplement 1.

| Experiment | Cell type | Receivers (clone) | Sender (clone) |
|---|---|---|---|
| Figure 4A | CHO-K1 | Notch1 (2D1) | Dll4-L1 |
| Figure 4A | CHO-K1 | Notch2 (2A4) | Dll1-L1 |
| Figure 4B; Figure 4-S1 | CHO-K1 | Notch1-H2B-mCh (F4) | Dll1-L2 |
| Figure 4B; Figure 4-S1 | CHO-K1 | Notch1-NGFR (2A6) | Dll1-L2 |
| Figure 4B; Figure 4-S1 | CHO-K1 | Notch1-Dll1 (2G5) | Dll1-L2 |
| Figure 4B; Figure 4-S1 | CHO-K1 | Notch1-Dll4 (1G11) | Dll1-L2 |
| Figure 4B; Figure 4-S1 | CHO-K1 | Notch1-Jag1 (1E2) | Dll1-L2 |
| Figure 4B; Figure 4-S1 | CHO-K1 | Notch1-Jag2 (2E10) | Dll1-L2 |
| Figure 4B; Figure 4-S1 | CHO-K1 | Notch2-H2B-mCh (G2) | Dll1-L2 |
| Figure 4B; Figure 4-S1 | CHO-K1 | Notch2-NGFR (1H4) | Dll1-L2 |
| Figure 4B; Figure 4-S1 | CHO-K1 | Notch2-Dll1 (1C4) | Dll1-L2 |
| Figure 4B; Figure 4-S1 | CHO-K1 | Notch2-Dll4 (1B8) | Dll1-L2 |
| Figure 4B; Figure 4-S1 | CHO-K1 | Notch2-Jag1 (2B8) | Dll1-L2 |
| Figure 4B; Figure 4-S1 | CHO-K1 | Notch2-Jag2 (1A1) | Dll1-L2 |
| Figure 6; Figure 6-S1 | CHO-K1 | Notch1-Dll1 (2G5) | Dll1-L2, Dll4-L2, Jag1-L1, Jag2-A |
| Figure 6; Figure 6-S1 | CHO-K1 | Notch1-Dll4 (1G11) | Dll1-L2, Dll4-L2, Jag1-L1, Jag2-A |
| Figure 6; Figure 6-S1 | CHO-K1 | Notch1-Jag1 (1E2) | Dll1-L2, Dll4-L2, Jag1-L1, Jag2-A |
| Figure 6; Figure 6-S1 | CHO-K1 | Notch1-Jag2 (2E10) | Dll1-L2, Dll4-L2, Jag1-L1, Jag2-A |
| Figure 6; Figure 6-S1 | CHO-K1 | Notch2-Dll1 (1C4) | Dll1-L2, Dll4-L2, Jag1-L1, Jag2-A |

bioRxiv preprint doi: https://doi.org/10.1101/2023.08.24.554677; this version posted August 25, 2023. The copyright holder for this preprint (which was not certified by peer review) is the author/funder, who has granted bioRxiv a license to display the preprint in perpetuity. It is made available under aCC-BY 4.0 International license.

| | | | |
|---|---|---|---|
| Figure 6; Figure 6-S1 | CHO-K1 | Notch2-Dll4 (1B8) | Dll1-L2, Dll4-L2, Jag1-L1, Jag2-A |
| Figure 6; Figure 6-S1 | CHO-K1 | Notch2-Jag1 (2B8) | Dll1-L2, Dll4-L2, Jag1-L1, Jag2-A |
| Figure 6; Figure 6-S1 | CHO-K1 | Notch2-Jag2 (1A1) | Dll1-L2, Dll4-L2, Jag1-L1, Jag2-A |
| Figure 7B | C2C12-Nkd | Notch1 (1A5) | Dll1 (c19) |
| Figure 7B | C2C12-Nkd | Notch2 (c24) | Dll1 (c19) |
| Figure 7C | C2C12-Nkd | Notch1-NGFR (1H6) | Dll4 (2H10) |
| Figure 7C | C2C12-Nkd | Notch1-Dll4 (2D1) | Dll4 (2H10) |
| Figure 7C | C2C12-Nkd | Notch1-Jag2 (1E7) | Dll4 (2H10) |
| Figure 7C | C2C12-Nkd | Notch2-NGFR (1E9) | Dll4 (2H10) |
| Figure 7C | C2C12-Nkd | Notch2-Dll4 (3B12) | Dll4 (2H10) |
| Figure 7C | C2C12-Nkd | Notch2-Jag2 (1B12) | Dll4 (2H10) |
| Figure 2A-C; Figure 2-S3; Figure 2-S1 | CHO-K1 | Notch1 (2D1); Notch2 (2A4) | Dll1-L4A |
| Figure 2A-C; Figure 2-S3; Figure 2-S1 | CHO-K1 | Notch1 (2D1); Notch2 (2A4) | Dll1-L1 |
| Figure 2A-C; Figure 2-S3; Figure 2-S1 | CHO-K1 | Notch1 (2D1); Notch2 (2A4) | Dll4-L4B |
| Figure 2A-C; Figure 2-S3; Figure 2-S1 | CHO-K1 | Notch1 (2D1); Notch2 (2A4) | Dll4-L1 |
| Figure 2A-C; Figure 2-S3; Figure 2-S1 | CHO-K1 | Notch1 (2D1); Notch2 (2A4) | Jag1-L3 |
| Figure 2A-C; Figure 2-S3; Figure 2-S1 | CHO-K1 | Notch1 (2D1); Notch2 (2A4) | Jag1-L1 |
| Figure 2A-C; Figure 2-S3; Figure 2-S1 | CHO-K1 | Notch1 (2D1); Notch2 (2A4) | Jag2-AB |
| Figure 2A-C; Figure 2-S3; Figure 2-S1 | CHO-K1 | Notch1 (2D1); Notch2 (2A4) | Jag2-A |
| Figure 2A-C only | CHO-K1 | Notch1-Dll1 (2G5) | Dll4-L4B |
| Figure 2A-C only | CHO-K1 | Notch1-Dll1 (2G5) | Jag2-A |
| Figure 2A-C only | CHO-K1 | Notch1-Dll4 (1G11) | Dll4-L4B |
| Figure 2A-C only | CHO-K1 | Notch1-Dll4 (1G11) | Jag2-A |

bioRxiv preprint doi: https://doi.org/10.1101/2023.08.24.554677; this version posted August 25, 2023. The copyright holder for this preprint (which was not certified by peer review) is the author/funder, who has granted bioRxiv a license to display the preprint in perpetuity. It is made available under aCC-BY 4.0 International license.

| | | | |
|---|---|---|---|
| Figure 2A-C only | CHO-K1 | Notch1-Jag1 (1E2) | Dll4-L4B |
| Figure 2A-C only | CHO-K1 | Notch1-Jag1 (1E2) | Jag2-A |
| Figure 2A-C only | CHO-K1 | Notch1-Jag2 (2E10) | Dll4-L4B |
| Figure 2A-C only | CHO-K1 | Notch1-Jag2 (2E10) | Jag2-A |
| Figure 2A-C only | CHO-K1 | Notch2-Jag1 (2B7); Notch2-Jag2 (1A1) | Dll4-L4B |
| Figure 2A-C only | CHO-K1 | Notch2-Jag1 (2B7); Notch2-Jag2 (1A1) | Dll1-L4A |
| Figure 2A-C only | CHO-K1 | Notch2-Jag1 (2B7); Notch2-Jag2 (1A1) | Jag2-B |
| Figure 2A-C only | CHO-K1 | Notch1 (2D1); Notch2 (2A4) | Dll4-L4 |
| Figure 2A-C only | CHO-K1 | Notch1 (2D1); Notch2 (2A4) | Jag1-L2 |
| Figure 2A-C only | CHO-K1 | Notch1 (2D1); Notch2 (2A4) | Jag2-AA |
| Figure 7A | C2C12-Nkd | Notch1 (1A5) | Dll1 (c19) |
| Figure 7A | C2C12-Nkd | Notch1 (1A5) | Jag1 (c11) |
| Figure 7A | C2C12-Nkd | Notch2 (c24) | Dll1 (c19) |
| Figure 7A | C2C12-Nkd | Notch2 (c24) | Jag1 (c11) |
| Figure 2D; Figure 2-S2 | CHO-K1 | Notch1 (2D1); Notch2 (2A4) | Tet-OFF Dll1 (G1) |
| Figure 2D; Figure 2-S2 | CHO-K1 | Notch1 (2D1); Notch2 (2A4) | Tet-OFF Dll4 (2D6) |
| Figure 2D; Figure 2-S2 | CHO-K1 | Notch1 (2D1); Notch2 (2A4) | Tet-OFF Jag1 (1G3) |
| Figure 2D; Figure 2-S2 | CHO-K1 | Notch1 (2D1); Notch2 (2A4) | Tet-OFF Jag2 (1H6) |
| Figure 5A; Figure 5-S4; Figure 5-S1; Figure 5-S2 | CHO-K1 | Notch1-Dll1 (2G5) | Dll4-L1 |
| Figure 5A; Figure 5-S4; Figure 5-S1; Figure 5-S2 | CHO-K1 | Notch1-Dll4 (1G11) | Dll4-L1 |
| Figure 5A; Figure 5-S4; Figure 5-S1; Figure 5-S2 | CHO-K1 | Notch1-Jag1 (1E2) | Dll4-L1 |
| Figure 5A; Figure 5-S4; Figure 5-S1; Figure 5-S2 | CHO-K1 | Notch1-Jag2 (2E10) | Dll4-L1 |

bioRxiv preprint doi: https://doi.org/10.1101/2023.08.24.554677; this version posted August 25, 2023. The copyright holder for this preprint (which was not certified by peer review) is the author/funder, who has granted bioRxiv a license to display the preprint in perpetuity. It is made available under aCC-BY 4.0 International license.

| | | | |
|---|---|---|---|
| Figure 5A; Figure 5-S4; Figure 5-S1; Figure 5-S2 | CHO-K1 | Notch2-Dll1 (1C4) | Dll1-L1 |
| Figure 5A; Figure 5-S4; Figure 5-S1; Figure 5-S2 | CHO-K1 | Notch2-Dll4 (1B8) | Dll1-L1 |
| Figure 5A; Figure 5-S4; Figure 5-S1; Figure 5-S2 | CHO-K1 | Notch2-Jag1 (2B7) | Dll1-L1 |
| Figure 5A; Figure 5-S4; Figure 5-S1; Figure 5-S2 | CHO-K1 | Notch2-Jag2 (1A1) | Dll1-L1 |

bioRxiv preprint doi: https://doi.org/10.1101/2023.08.24.554677; this version posted August 25, 2023. The copyright holder for this preprint (which was not certified by peer review) is the author/funder, who has granted bioRxiv a license to display the preprint in perpetuity. It is made available under aCC-BY 4.0 International license.

## Supplementary Table 5: Recombinant Notch Ligands and Concentrations

| Experiment | Cell type | Receivers (clone) | Ligand (conc.) |
|---|---|---|---|
| Figure 7-S1A (plated ligand assay) | C2C12 | C2C12 (wt) | Dll1-ext-Fc* (2 μg/mL) |
| Figure 7-S1A (plated ligand assay) | C2C12 | C2C12 (Nkd) | Dll1-ext-Fc* (2 μg/mL) |
| Figure 3G,H (soluble ligand binding assay) | CHO-K1 | Parental reporter (cH1); Notch1 (2D1); Notch2 (2A4) | Dll1-ext-Fc (1 nM) |
| Figure 3G,H (soluble ligand binding assay) | CHO-K1 | Parental reporter (cH1); Notch1 (2D1); Notch2 (2A4) | Dll4-ext-Fc (1 nM) |
| Figure 3G,H (soluble ligand binding assay) | CHO-K1 | Parental reporter (cH1); Notch1 (2D1); Notch2 (2A4) | Jag1-ext-Fc (0.5 nM) |
| Figure 3G,H (soluble ligand binding assay) | CHO-K1 | Parental reporter (cH1); Notch1 (2D1); Notch2 (2A4) | Jag2-ext-Fc (1 nM) |
| Figure 3I (plated ligand assay) | CHO-K1 | Notch1 (2D1) | Dll1-ext-Fc (75 and 150 nM) |
| Figure 3I (plated ligand assay) | CHO-K1 | Notch1 (2D1) | Dll4-ext-Fc (50 nM) |
| Figure 3I (plated ligand assay) | CHO-K1 | Notch1 (2D1) | Jag1-ext-Fc (20 and 50 nM) |
| Figure 3I (plated ligand assay) | CHO-K1 | Notch1 (2D1) | IgG1 Fc (150 nM) |
| Figure 3B; Figure 3-S1 (plated ligand assay) | CHO-K1 | Notch1 (2D1); Notch2 (2A4) | Dll1-ext-Fc (0.5, 1, 2, 3, 5, 10, 15, 25, 50, and 100 nM) |
| Figure 3B; Figure 3-S1 (plated ligand assay) | CHO-K1 | Notch1 (2D1) | Dll4-ext-Fc (0.1, 0.5, 1, 2, 2.5, 3, 5, 10, and 15 nM) |
| Figure 3B; Figure 3-S1 (plated ligand assay) | CHO-K1 | Notch1 (2D1) | Jag1-ext-Fc (0.5, 2, 3, 5, 10, 15, 25 and 50 nM) |
| Figure 3B; Figure 3-S1 (plated ligand assay) | CHO-K1 | Notch1 (2D1); Notch2 (2A4) | Jag2-ext-Fc (0.5, 1, 2, 3, 5, 10, 15, 25, 50, and 100 nM) |
| Figure 3B; Figure 3-S1 (plated ligand assay) | CHO-K1 | Notch1 (2D1); Notch2 (2A4) | IgG1 Fc (0.5, 2, 3, 5, 10, 15, 25, 50, and 100 nM) |

bioRxiv preprint doi: https://doi.org/10.1101/2023.08.24.554677; this version posted August 25, 2023. The copyright holder for this preprint (which was not certified by peer review) is the author/funder, who has granted bioRxiv a license to display the preprint in perpetuity. It is made available under aCC-BY 4.0 International license.

| | | | |
|---|---|---|---|
| Figure 3B; Figure 3-S1 (plated ligand assay) | CHO-K1 | Notch2 (2A4) | Dll4-ext-Fc (0.1, 0.5, 1, 2, 5, 10, 15, 25, 50, and 100 nM) |
| Figure 3B; Figure 3-S1 (plated ligand assay) | CHO-K1 | Notch2 (2A4) | Jag1-ext-Fc (0.5, 2, 3, 5, 10, 15, and 25 nM) |

*Dll1-ext-Fc provided by Irwin Bernstein; all other recombinant ligands are from Bio-Techne/R&D Systems (see Key Resources Table)

bioRxiv preprint doi: https://doi.org/10.1101/2023.08.24.554677; this version posted August 25, 2023. The copyright holder for this preprint (which was not certified by peer review) is the author/funder, who has granted bioRxiv a license to display the preprint in perpetuity. It is made available under aCC-BY 4.0 International license.

**Supplementary Table 6: Primers for RT-PCR and qRT-PCR**

| PCR Method | Gene Target | Forward Primer | Reverse Primer |
|---|---|---|---|
| RT-PCR | Mouse Jag1 | CCAAAGCCTCTCAACTTAGTGC | CTTAGTTTTCCCGCACTTGTGTTT |
| qRT-PCR | mCherry | CCTCAGTTCATGTACGGCTCC | TCGAAGTTCATCACGCGCTC |
| qRT-PCR | Mouse Notch1 | ATGTCAATGTTCGAGGACCAG | TCACTGTTGCCTGTCTCAAG |
| qRT-PCR | Mouse Notch2 | GGACTGTGAAGAGGACATCAAT | CACTGGCAGGAGAAGGTATTC |
| qRT-PCR | Mouse Notch3 | TGCCAGGGAATTTCAGGTG | AGGCAAGAACAGGAAAAGGAG |
| qRT-PCR | Mouse SdhA (housekeeping) | AGTGGGCTGTCTTCCTTAAC | GGATTGCTTCTGTTTGCTTGG |
| qRT-PCR | CHO Lfng | GAGGAGCTGGCTTCTGTATTAG | AGCAGTGCTTCCACAATGTA |
| qRT-PCR | CHO or Mouse Rfng | GTCCTGAGAACCCACGTAATG | AGTGGGTGTCTGGGTAGAGAA |
| qRT-PCR | CHO Beta-actin (housekeeping) | ACTGGGACGATATGGAGAAG | GGTCATCTTTTCACGGTTGG |
